# Interferon regulatory factor 8 regulates caspase-1 expression to facilitate Epstein-Barr virus reactivation in response to B cell receptor stimulation and chemical induction

**DOI:** 10.1371/journal.ppat.1006868

**Published:** 2018-01-22

**Authors:** Dong-Wen Lv, Kun Zhang, Renfeng Li

**Affiliations:** 1 Department of Oral and Craniofacial Molecular Biology and Philips Institute for Oral Health Research, School of Dentistry, Virginia Commonwealth University, Richmond, Virginia, United States of America; 2 Department of Microbiology and Immunology, School of Medicine, Virginia Commonwealth University, Richmond, Virginia, United States of America; 3 Massey Cancer Center, Virginia Commonwealth University, Richmond, Virginia, United States of America; Baylor College of Medicine, UNITED STATES

## Abstract

Interferon regulatory factor 8 (IRF8), also known as interferon consensus sequence-binding protein (ICSBP), is a transcription factor of the IRF family. IRF8 plays a key role in normal B cell differentiation, a cellular process that is intrinsically associated with Epstein-Barr virus (EBV) reactivation. However, whether IRF8 regulates EBV lytic replication remains unknown. In this study, we utilized a CRISPR/Cas9 genomic editing approach to deplete *IRF8* and found that *IRF8* depletion dramatically inhibits the reactivation of EBV upon lytic induction. We demonstrated that *IRF8* depletion suppresses the expression of a group of genes involved in apoptosis and thus inhibits apoptosis induction upon lytic induction by B cell receptor (BCR) stimulation or chemical induction. The protein levels of caspase-1, caspase-3 and caspase-8 all dramatically decreased in *IRF8*-depleted cells, which led to reduced caspase activation and the stabilization of KAP1, PAX5 and DNMT3A upon BCR stimulation. Interestingly, caspase inhibition blocked the degradation of KAP1, PAX5 and DNMT3A, suppressed EBV lytic gene expression and viral DNA replication upon lytic induction, suggesting that the reduced caspase expression in *IRF8*-depleted cells contributes to the suppression of EBV lytic replication. We further demonstrated that IRF8 directly regulates *CASP1 (caspase-1)* gene expression through targeting its gene promoter and knockdown of caspase-1 abrogates EBV reactivation upon lytic induction, partially through the stabilization of KAP1. Together our study suggested that, by modulating the activation of caspases and the subsequent cleavage of KAP1 upon lytic induction, IRF8 plays a critical role in EBV lytic reactivation.

## Author summary

Infection with Epstein-Barr virus (EBV) is closely associated with human cancers of both B cell and epithelial cell origin. The EBV life cycle is tightly regulated by both viral and cellular factors. Here, we demonstrate that interferon regulatory factor 8 (IRF8) is required for EBV lytic replication. Mechanistically, IRF8 directly regulates caspase-1 expression and hence caspase activation upon B cell receptor (BCR) stimulation and chemical induction, which leads to the cleavage and de-stabilization of several host factors suppressing lytic replication, including KAP1. *Caspase-1* depletion blocks EBV reactivation while *KAP1* depletion facilitates reactivation in *caspase*-1 depleted cells. These results together establish a IRF8/caspase-1/KAP1 axis important for EBV reactivation.

## Introduction

Epstein-Barr virus (EBV), a ubiquitous human gammaherpesvirus, is associated with malignant diseases, including Burkitt’s lymphoma, Hodgkin’s lymphoma, nasopharyngeal carcinoma, and NK/T cell lymphoma [1]. The genome of EBV is approximately 170 kb in length and encodes more than 80 genes. EBV infects both B lymphocytes and some epithelial cells and the life cycle of EBV is divided into latent or lytic phases. In the lytic phase, EBV expresses all lytic genes and progeny virus particles are packaged and released from the cell [2]. The reactivation of EBV from latent to lytic phase can be triggered by expression of two viral immediate-early gene products, ZTA (also called BZLF1 or Z) and RTA (also known as BRLF1 or R). A series of cellular factors have been shown to regulate *ZTA* and *RTA* gene expression and to affect ZTA/RTA transcriptional activity [3,4,5,6,7,8,9,10,11,12,13,14,15,16]. B cell receptor (BCR) activation is a philologically relevant stimulus for triggering EBV reactivation from latency since this occurs not only in tumor cell lines but also in freshly isolated B cells from patients [17,18].

The interferon regulatory factor (IRF) family members (IRF1-9) are transcription factors for interferon (IFN) and IFN-inducible genes [19,20]. Members of the IRF family also play a vital role in regulation of immunity and oncogenesis [21]. Previous studies showed that several IRFs are implicated in the life cycles of herpesviruses, including EBV. For examples, IRF1, IRF2, IRF4, IRF5 and IRF7 are involved in EBV latency and virus-mediated cell transformation [22,23,24,25,26]. IRF4 synergizes with RTA encoded by murine γ-herpesvirsus-68 to facilitate viral *M1* gene expression [27]. IRF3 and IRF7-mediated antiviral responses are counteracted by EBV encoded proteins [28,29,30].

IRF8, also known as IFN consensus sequence-binding protein (ICSBP), is a unique transcription factor of the IRF family because it is expressed predominately in hematopoietic cells [31]. Similar to other IRFs, IRF8 contains a DNA binding domain (DBD) and interacts with other proteins (such as PU.1, IRF1, IRF2 or IRF4) through the IRF association domain (IAD). In addition, IRF8 can be tyrosine phosphorylated [32,33,34,35], SUMOylated [36] and ubiquitinated [37,38]. The DBD, IAD and post-translational modifications of IRF8 all contribute to its transcription-regulatory activities [36,39,40,41]. Phosphorylation and dephosphorylation can alter the function of IRF8 in innate immune responses and leukemia pathogenesis [34,42]. SUMO conjugation-deconjugation switches IRF8’s function as a repressor or a activator [36]. IRF8 is ubiquitinated by an E3 ligase TRIM21, which alters IRF8’s ability in *IL12p40* transcription [30,37]. Knockdown of IRF8 inhibits the growth of diffuse large B-cell lymphoma [43]. IRF8 is required for apoptotic induction in myeloid cells [44]. Recently, an important study established a role for IRF4 and IRF8 in EBV-mediated B-cell transformation [45]. EBV EBNA3C, which is expressed in cells of type III latency, interacts with and stabilizes IRF4. EBNA3C coordinates with IRF4 to downregulate IRF8, which is critical for apoptosis inhibition and thus the survival of EBV-transformed cells [45]. However, in EBV-positive B cells of type I latency, EBNA3C is not expressed and IRF4 protein level is very low while IRF8 is highly expressed [46]. Despite the high expression of IRF8 in B cells of type I EBV latency, the contribution of IRF8 to EBV lytic replication remains unknown.

Driven by these facts, we explored the role of IRF8 in the EBV lytic cycle. We demonstrated that IRF8 positively regulates EBV lytic replication through regulating caspases expression and hence caspase activation upon lytic induction and caspase activation facilitates the degradation of cellular factors that limit EBV lytic replication.

## Results

### *IRF8* depletion suppresses EBV lytic replication

The previous research on IRF8 and EBV latency [45] and the high expression of IRF8 in EBV-positive B cells of type I latency prompted us to test whether and how IRF8 regulates EBV lytic replication. Here we first utilized an Akata (EBV^+^) cell line, a Burkitt’s lymphoma cell line of type I latency, as a model system to investigate the role of IRF8 in the EBV lytic cycle. Because Akata (EBV^+^) cells express surface immunoglobulin receptors of the G (κ) class (IgG) and anti-IgG cross-linking mediated BCR activation can serve as a physiologically relevant stimulus for EBV lytic reactivation [18], these cells are well-suited for investigating the contribution of cellular factors in EBV lytic replication [13,47].

**Fig 1:**
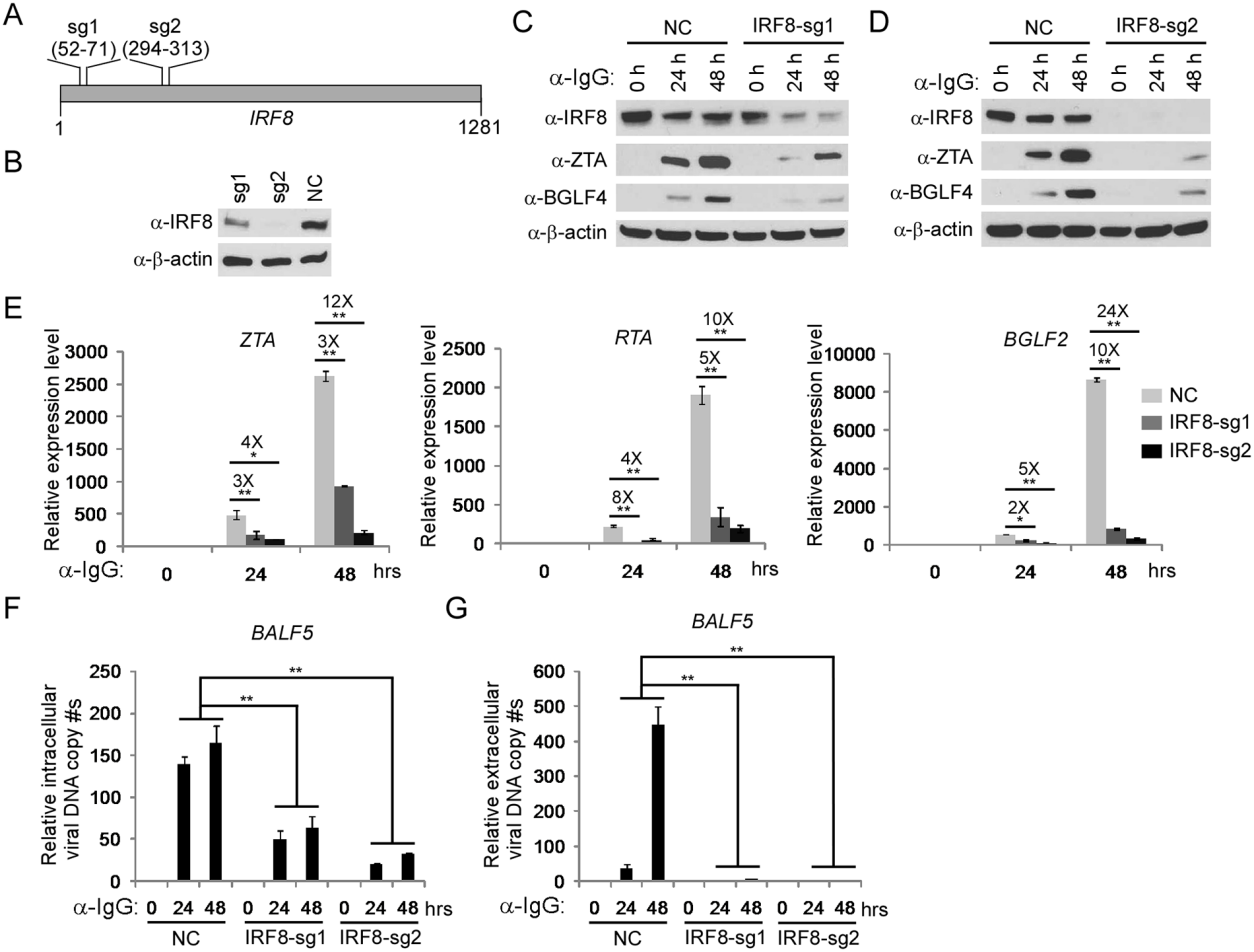
*IRF8* depletion inhibits the reactivation of EBV in Akata (EBV^+^) cells. A. The locations of two sgRNAs (sg1 and sg2) used for *IRF8* depletion. B. Western blot was performed to check the knockdown efficiency of IRF8 by sg1 and sg2 compared with the non-targeting control sgRNA (NC). C-G.*IRF8*-depleted (sg1 and sg2) and control (NC) Akata (EBV^+^) cells were either untreated (0 hr) or treated with anti-IgG for 24 and 48 hrs to induce lytic replication. The cell pellets and supernatant was harvested 24 and 48 hrs after anti-IgG stimulation. Protein extracts were analyzed by western blot using antibodies against IRF8 and EBV immediate-early (ZTA) and early (BGLF4) proteins (C and D). RT-qPCR showing the suppression of EBV immediate-early (*ZTA* and *RTA*) and late (*BGLF2*) genes expression upon IRF8 depletion (sg1 and sg2) (E). qPCR showing the reduction of intracellular viral DNA (F) and extracellular virion-associated DNA (G) copy numbers upon IRF8 depletion. The EBV genome copy number was measured by qPCR using primers specific to EBV *BALF5*. The intracellular EBV copy number was normalized by qPCR using specific primers to *β-actin*. Data are presented as means ± standard deviations (*n* = 3). * p<0.05 and ** *p*<0.01.

To demonstrate whether IRF8 regulates EBV lytic replication, we utilized CRISPR/Cas9 technology to knockdown endogenous *IRF8* in Akata (EBV^+^) B cells. We designed two sgRNAs and used a lenti-viral system to establish two *IRF8*-depleted pool cell lines (Fig 1A). To ensure the reproducibility of our results, at least three independent lentiviral infections were performed. The infection efficiency was approximately 20% and the experiments were performed after one to two weeks selection with puromycin when all living cells were puromycin-resistant. Compared with non-targeting control (NC), the sgRNA sg1 partially knocked down the protein expression of IRF8, while sg2 efficiently depleted IRF8 (Fig 1B). To further confirm the correct targeting of *IRF8* by CRISPR/Cas9, we sequenced the genomic DNA spanning the CRISPR/Cas9 targeting region of the *IRF8-sg1* and *IRF8-sg2* cell lines and we found that 10 out of 22 clones for sg1 and 9 out of 14 clones for sg2 contain frame shifts (S1 Fig). To evaluate the effects of *IRF8* depletion on EBV lytic replication, we triggered EBV lytic replication by anti-IgG mediated BCR cross-linking. We found that the accumulation of the EBV lytic proteins ZTA and BGLF4 was suppressed in the two *IRF8*-depleted cell lines upon lytic induction and that the higher IRF8 knockdown efficiency correlated with lower ZTA and BGLF4 expression (Fig 1C and 1D).

We then examined the level of lytic RNA transcripts in these cell lines. As expected, knockdown of IRF8 dramatically suppressed the expression of immediate early (*ZTA* and *RTA*) and late (*BGLF2*) genes (Fig 1E). To test whether IRF8 plays a role in EBV replication, we measured both intracellular and extracellular EBV genome copies following lytic induction. We found that both intracellular (Fig 1F) and extracellular (Fig 1G) viral DNA copies were significantly reduced upon IRF8 depletion. These results suggested that IRF8 acts as a key positive regulator during EBV lytic reactivation.

To further demonstrate that the observed phenotype was not due to off-target effects, we reconstituted *IRF8* back into the *IRF8*-depleted (sg2) cells. We found that *IRF8* restoration facilitated EBV ZTA and RTA protein expression compared with *IRF8*-depleted cells upon IgG cross-linking (S2A Fig, lanes 2–3 vs 5–6). Moreover, EBV DNA replication was also dramatically enhanced upon *IRF8* reconstitution (S2B Fig, lanes 2–3 vs 5–6). Together these results suggest that IRF8 promotes EBV replication upon lytic induction.

### IRF8-dependent caspase activation is required for EBV reactivation

**Fig 2:**
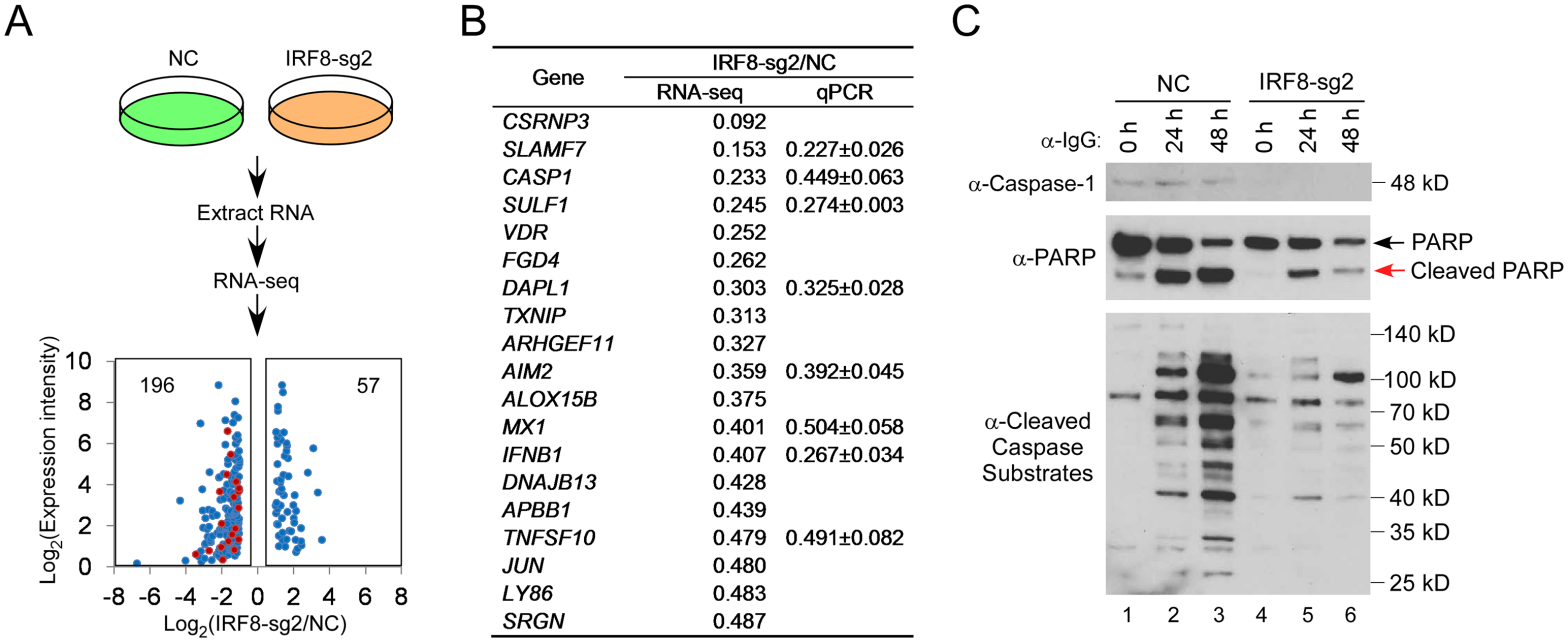
*IRF8* depletion suppresses the expression of genes involved in apoptosis. A. Schematic representation of RNA-seq analyses of Akata (EBV^+^) cells carrying control (NC) or *IRF8-sg2* sgRNAs, RNAs were extracted from cells derived from three distinct lentiviral transductions. Using 2-fold change as a cutoff, 196 and 57 genes were down- or up-regulated upon *IRF8* depletion, respectively. Gene Ontology analysis showing that 19 genes involved in “positive regulation of apoptosis” (red dots) were down-regulated by IRF8 depletion. B. Fold changes of the 19 apoptosis-related genes and the validation of 8 of them by RT-qPCR analysis of RNAs from cells derived from three distinct lentiviral transductions. C. *IRF8* depletion (sg2) suppresses caspase-1 expression and the generation of cleaved caspase substrates upon lytic induction by anti-IgG cross-linking. Western blot analysis of protein extracts from Fig 1D using antibodies against caspase-1, PARP, and cleaved caspase substrates (Peptides containing [DE(T/S/A)D] motif) as indicated.

As a transcription factor, IRF8 may also regulate EBV replication through altering cellular processes. To provide insight into IRF8-regulated cellular events, we performed RNA-Seq analysis for the control and *IRF8*-depleted cells generated from three different lentiviral transductions. Totally we identified 253 differentially expressed genes (S1 Table). Among these genes, 196 genes were down-regulated and 57 genes were up-regulated upon IRF8 depletion (Fig 2A). Gene Ontology (GO) analysis plus manual curation of these differentially regulated genes revealed that 19 genes involved in “positive regulation of apoptosis” were significantly enriched. Interestingly, all of these genes involved in apoptosis were down-regulated in IRF8-depleted cells (Fig 2A, red dots and Fig 2B). To validate our RNA-seq results, we selected 8 genes and analyzed their expression by RT-qPCR for both *IRF8-sg1* and *IRF8-sg2* cells. The down-regulation was confirmed for all those genes tested, including *caspase-1* (*CASP1*) (Figs 2B and S3A). Consistent with the reduced mRNA expression, caspase-1 protein level was reduced in *IRF8*-depleted cells (Figs 2C and S3B, lane 1 vs 4). The down-regulation of apoptosis related genes suggested that IRF8 depletion may suppress apoptosis induction during EBV lytic replication upon BCR activation. To test this possibility, we monitored the cleavage of PARP and global caspase substrates containing a cleavage motif [DE(T/S/A)D]. We found that IRF8 depletion suppressed protein cleavage upon BCR activation (Figs 2C and S3B, lanes 2–3 vs 5–6).

**Fig 3:**
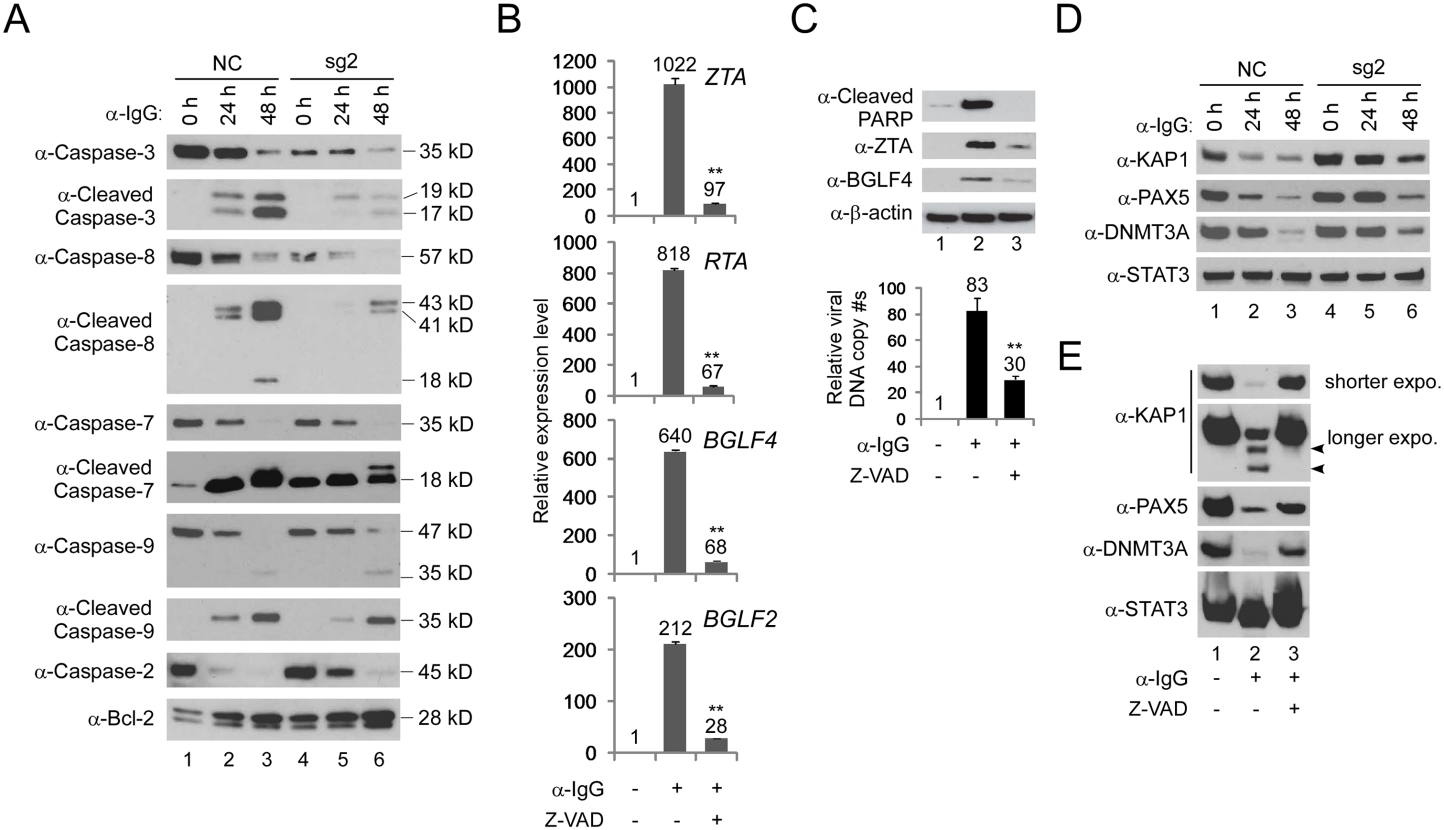
*IRF8* depletion suppresses caspase activation and caspase activation is required for EBV lytic replication. A. *IRF8* depletion suppresses caspase activation. Western blot analysis of protein extracts from Fig 1D using antibodies against caspase-3, cleaved caspase-3, caspase-8, cleaved caspase-8, caspase-7, cleaved caspase-7, caspase-9, cleaved caspass-9, caspase-2 and Bcl-2 as indicated. B. Caspase inhibition suppresses EBV lytic gene expression. Akata (EBV^+^) cells were untreated or pre-treated with pan-caspase inhibitor (Z-VAD-FMK) for 1 hr and then anti-IgG was added for 48 hrs. RNA was extracted and EBV lytic gene expression was analyzed by RT-qPCR. Data are presented as means ± standard deviations of triplicate assays. ** p<0.01 (compared with the second bar). C. Caspase inhibition suppresses EBV DNA replication. Protein extracts from cells treated as Panel B were analyzed by western blot using antibodies against cleaved-PARP, EBV ZTA and BGLF4 as indicated. β-actin was used as loading controls. Genomic DNA was extracted and relative EBV DNA copy numbers was measured by qPCR using primers specific to EBV *BALF5*. The EBV copy number was normalized by qPCR using specific primers to *β-actin*. Data are presented as means ± standard deviations of triplicate assays. ** p<0.01 (compared with the second bar). D. IRF8 depletion suppresses the degradation of KAP1, PAX5 and DNMT3A upon lytic induction. Western blot analysis of protein extracts from Fig 1D using antibodies against KAP1, PAX5, DNMT3A and STAT3 as indicated. E. Caspase inhibition restores the expression of KAP1, PAX5 and DNMT3A. Protein extracts from Panel C were analyzed by western blot using antibodies against KAP1, PAX5, DNMT3A and STAT3 as indicated. The longer exposure of KAP1 blot revealed two cleaved KAP1 products upon lytic induction (lane 2, arrow heads).

IRF8 has been shown to positively regulate the apoptosis of myeloid cells and nonhematopoietic tumor cells [44,48,49,50]. The dramatic down-regulation of caspase-mediated protein cleavage upon IRF8 depletion suggested that IRF8 may regulate the activation of caspases. To test this possibility, we monitored the level of individual caspases and their cleaved products. Strikingly, we found that the IRF8 depletion markedly reduced the levels of caspase-3 and caspase-8 and consequently the generation of active cleaved products was also suppressed upon BCR activation. In contrast, the protein levels of caspase-2, caspase-7 and caspase-9 and their cleavage were less affected by IRF8 depletion (Figs 3A and S3C). In addition, the level of Bcl-2, an anti-apoptosis protein, increased in *IRF8*-depleted cells (Figs 3A and S3C, Bcl-2 blot, lanes 1–3 vs 4–6), which further contributed to IRF8-dependent inhibition of apoptosis. Except for *caspase-1*, the gene expression levels of other caspases, including *caspase-3* and *caspase-8*, were not regulated by IRF8 depletion according to our RNA-seq analysis (S4 Fig and S1 Table), suggesting that IRF8 may control caspase-3 and caspase-8 protein levels through modulation of translation or protein stability rather than transcription.

Because caspase activation upon apoptotic induction can facilitate EBV lytic reactivation in other EBV-positive cell lines [51,52], we reasoned that IRF8 facilitates EBV reactivation in the Akata (EBV^+^) cells through caspase activation. To test this hypothesis, we pretreated the Akata (EBV^+^) cells with a pan-caspase inhibitor Z-VAD-FMK and then induced EBV lytic reactivation by anti-IgG cross-linking of the BCR. Caspase inhibition strongly suppressed the expression immediate-early (*ZTA* and *RTA*), early (*BGLF4*) and late (*BGLF2*) gene expression (Fig 3B). Consistently, the EBV ZTA and BGLF4 protein expression and viral DNA replication were also blocked by caspase inhibition (Fig 3C).

The switch from EBV latency to lytic reactivation is negatively regulated by a number of cellular factors [53]. Because caspase activation can lead to the cleavage of many cellular proteins [54,55,56], we hypothesized that those factors normally suppressing EBV lytic replication are destabilized by caspase activation upon BCR stimulation. To test this hypothesis, we monitored the levels of several proteins, including KAP1 [12,57,58,59], PAX5 [8,60,61,62], DNMT3A [63] and STAT3[64,65,66,67,68], whose functions have been shown to maintain herpesviruses latency and suppress lytic replication/reactivation. We found that the protein levels of KAP1, PAX5 and DNMT3A, but not that of STAT3, were dramatically reduced upon lytic induction (Figs 3D and S3C, lanes 1–3) while IRF8 depletion suppressed the down-regulation of KAP1, PAX5 and DNMT3A (Figs 3D and S3C, lanes 4–6). To further test whether caspase activation plays a role in the de-stabilization of KAP1, PAX5 and DNMT3A, we monitored their protein levels in Akata (EBV^+^) cells when caspases are inhibited and lytic replication is triggered by BCR stimulation. Interestingly, pretreatment of the cells with a pan-caspase inhibitor Z-VAD-FMK restored their expression (Fig 3E). For KAP1, in addition to the reduced protein level, we also noticed the generation of two potential cleaved fragments upon BCR activation, which is also blocked by caspase inhibition (Fig 3E, KAP1, longer exposure). Taken together, these results suggested caspase activation-mediated de-stabilization of cellular restriction factors contributes to EBV lytic replication.

**Fig 4:**
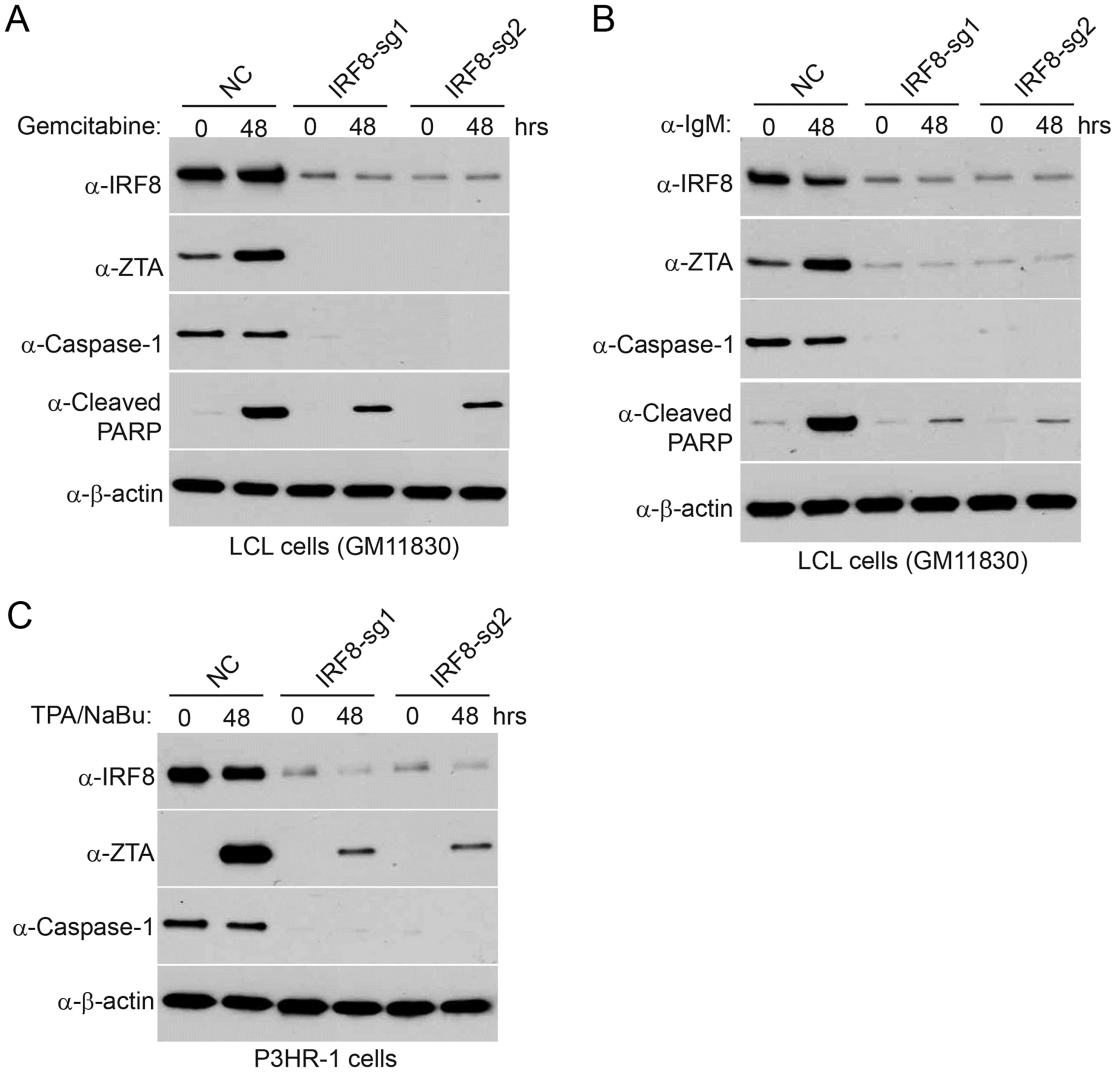
*IRF8* depletion suppresses EBV reactivation in LCL and P3HR-1 cells upon lytic induction. A and B. Control (NC) and *IRF8*-depleted (sg1 and sg2) LCL cells were either untreated (0 hr) or treated with 1 μg/mL gemcitabine (A) or 20 μg/mL α-IgM (B) for 48 hrs to induce lytic replication. Western blot analyses showing IRF8, ZTA, caspase-1 and cleaved-PARP level as indicated. C. Control (NC) and *IRF8*-depleted (sg1 and sg2) P3HR-1 cells were either untreated (0 hr) or treated with TPA (20 ng/ml)/sodium butyrate (NaBu, 3 mM) for 48 hrs to induce lytic replication. Western blot analyses showing IRF8, ZTA and caspase-1 level as indicated.

To demonstrate the effect of IRF8 in additional EBV-positive cell lines, we depleted *IRF8* in two additional cell lines, P3HR-1 and an EBV transformed lymphoblastoid cell line (LCL). We observed universal lower reactivation for EBV in IRF8-depleted cells treated with either gemcitabine, anti-IgM (for LCL cells) or TPA/sodium butyrate (for P3HR-1 cells) (Fig 4), reinforcing that IRF8 plays a key role in EBV reactivation.

### IRF8 regulates *CASP1 (caspase-1)* promoter activity

**Fig 5:**
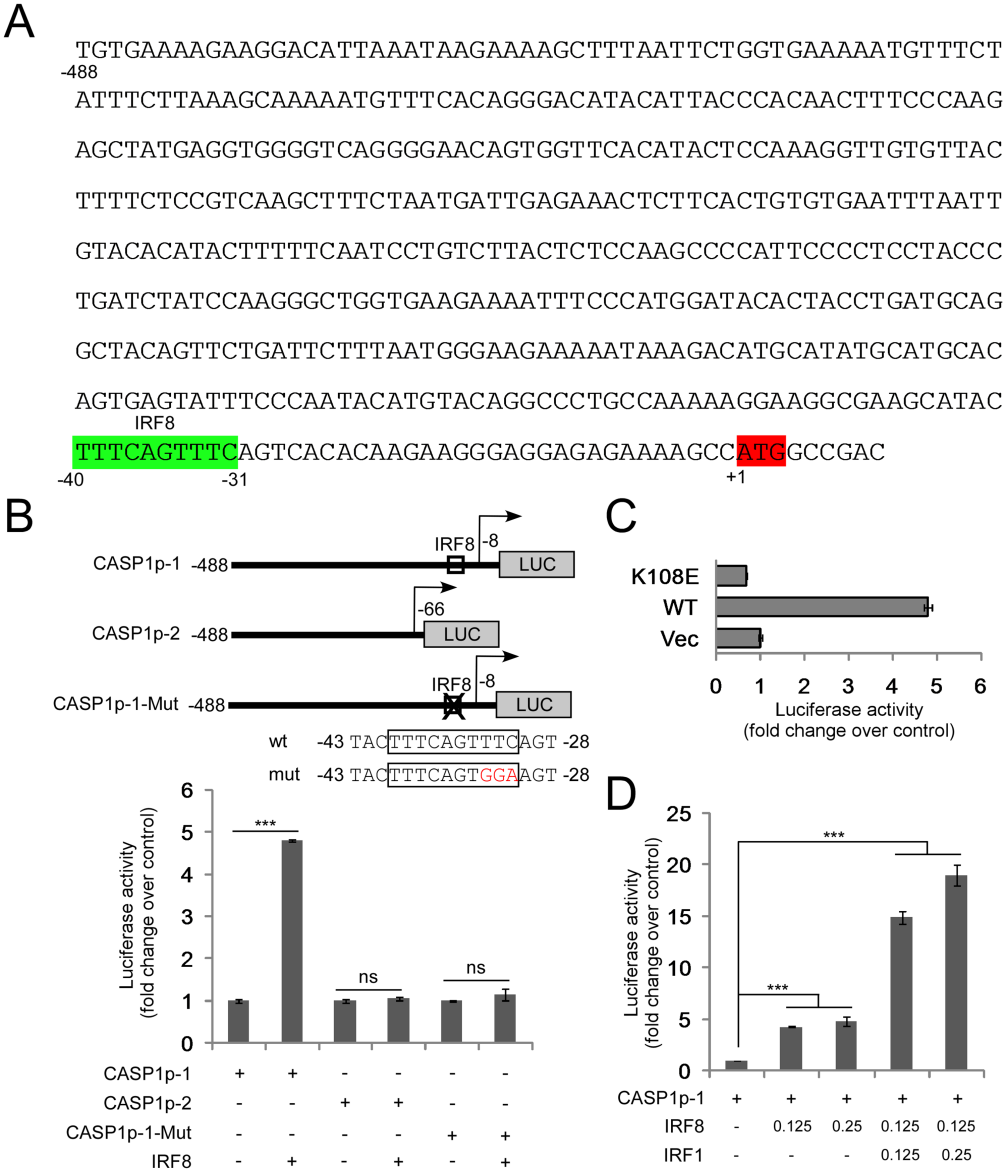
IRF8 regulates *CASP1* promoter activities. A. Schematic representation of the promoter of human *CASP1*. IRF8 consensus binding site is highlighted in green. The ATG of *CASP1* is highlighted in red. B. The pGL2-CASP1p constructs (with or without IRF8 consensus site) and the IRF8 consensus site mutated construct were co-transfected into 293T cells with either vector control or IRF8 expression vectors. Luciferase assays were performed 36 hrs post-transfection. The value of cells transfected with empty vectors was set as 1. The results were presented as mean ± standard deviation of triplicate assays. C. The pGL2-CASP1p1 construct was co-transfected into 293T cells with either vector control, wild-type IRF8 (WT) or IRF8 DNA binding mutant (K108E) expression vectors and luciferase assays were performed 36 hrs post-transfection. The value of cells transfected with empty vectors was set as 1. The results were presented as mean ± standard deviation of triplicate assays. D. The pGL2-CASP1p1 construct was co-transfected into 293T cells with either vector control or IRF8 and IRF1 expression vectors and luciferase assays were performed 36 hrs post-transfection. The value of cells transfected with empty vectors was set as 1. The results were presented as mean ± standard deviation of triplicate assays. *** p<0.001.

Based on our RNA-seq results, only *CASP1 (caspase-1)* gene was regulated by IRF8 at the RNA level (S4 Fig). Previous studies using ChIP-seq showed that IRF8 could bind to the promoter regions of both human and mouse *CASP1* at a conserved consensus site, -40 to -31 bp upstream of the start codon of human *CASP1* (Fig 5A) [69,70]. However, it is not clear whether IRF8 directly regulates *CASP1* expression. We hypothesized that IRF8, as a transcription activator, directly regulates *CASP1* gene expression through binding to its promoter. To test our hypothesis, we constructed luciferase reporter plasmids, which contain the *CASP1* promoter with or without the putative IRF8 binding site (Fig 5B). The luciferase reporter assay showed that IRF8 activated the wild-type *CASP1* promoter but not the truncated version without the IRF8 binding site (Fig 5B). To confirm our results, we mutated the conserved IRF8 binding site and found that IRF8 failed to activate the mutated reporter (Fig 5B). To further validate our results, we constructed a DNA-binding deficient IRF8 mutant (K108E) [71] and tested whether it can block the activation of *CASP1* promoter. Compared with wild-type IRF8, the DNA-binding deficient mutant (K108E) lost the ability to regulate the *CASP1* promoter (Fig 5C). In conclusion, our results demonstrated that IRF8 enhances *CASP1* gene expression through regulation of its promoter. A previous study showed that IRF1 can also regulate *CASP1* gene promoter [72]. Therefore, we tested whether IRF1 could cooperate with IRF8 to further enhance the *CASP1* promoter activity. The luciferase reporter assay demonstrated that IRF1 synergized with IRF8 to further enhance *CASP1* promoter activity (Fig 5D).

To further prove whether IRF8/IRF1 bind to *CASP1* promoter in B cells, we performed ChIP experiments using chromatin prepared from EBV-postive Akata, LCL and P3HR-1 cells. Our results showed that IRF8/IRF1 indeed bind to the promoter region of *CASP1* for all these cells (S5A Fig), suggesting that they directly regulate *CASP1* expression *in vivo*. To demonstrate physiological relevance of IRF8/IRF1 activation of *CASP1* promoter observed in 293T cells, we performed luciferase assay using Akata cells. Similarly, we found that IRF8 and IRF1 triggered a strong activation of *CASP1* promoter while the IRF8 DNA binding deficient mutant (K108E) failed to activate the promoter (S5B Fig).

Our RNA-seq analysis showed that both *IRF1* and *IRF8* are expressed in the Akata (EBV^+^) cells, with *IRF8* level approximately 6-fold higher than that of *IRF1* (S6 Fig). Based on the luciferase assay, IRF8, together with its closely related family member IRF1, plays an effective role on regulating *CASP1* expression.

### *Caspase*-1 depletion abrogates EBV lytic replication

**Fig 6:**
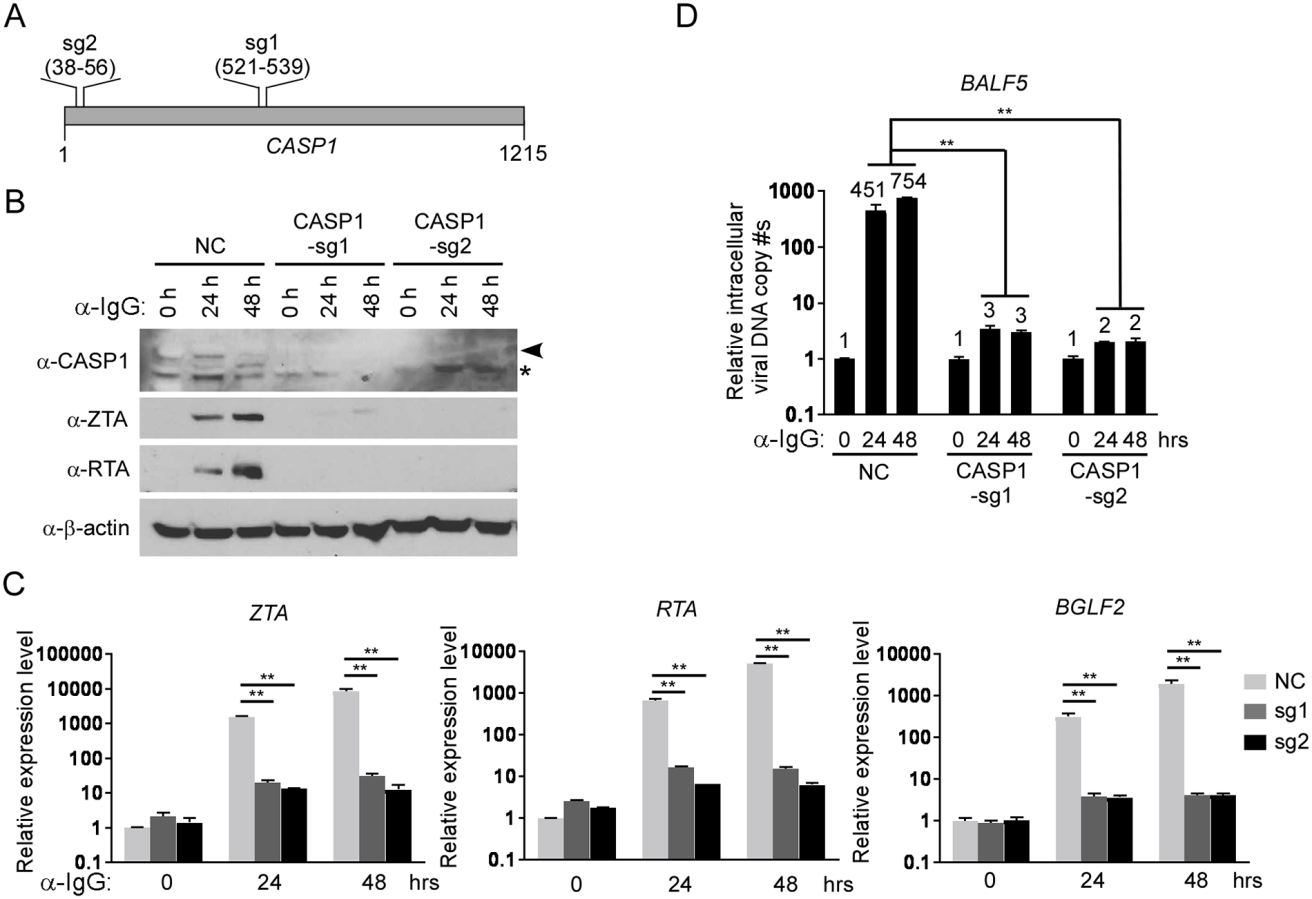
*CASP1* depletion inhibits the reactivation of EBV in Akata (EBV^+^) cells. A. The locations of two sgRNAs (sg1 and sg2) used for CASP1 depletion. B-D. *CASP1*-depleted (sg1 and sg2) and control (NC) Akata (EBV^+^) cells were either untreated (0 hr) or treated with anti-IgG for 24 and 48 hrs to induce lytic replication. The cell pellets were harvested 24 and 48 hrs after anti-IgG stimulation. Protein extracts were analyzed by western blot using antibodies against CASP1 and EBV immediate-early (ZTA and RTA) proteins and β-actin (B). RT-qPCR showing the suppression of EBV immediate-early (*ZTA* and *RTA*) and late (*BGLF2*) genes expression upon CASP1 depletion (sg1 and sg2) (C). qPCR showing the reduction of intracellular viral DNA copy numbers upon CASP1 depletion (D). The EBV genome copy number was measured by qPCR using primers specific to EBV *BALF5*. The intracellular EBV copy number was normalized by qPCR using specific primers to *β-actin*. Data are presented as means ± standard deviations (*n* = 3). ** *p*<0.01.

The control of *caspase-1* expression by IRF8 promoted us to test whether caspase-1 contributes to EBV reactivation upon lytic induction. To answer this question, we utilized a similar CRISPR/Cas9 approach to deplete endogenous *CASP1* in Akata (EBV^+^) B cells. To offset the potential off-target effect, we designed two sgRNAs to establish *CASP1*-depleted cell lines by three distinct lentiviral infections (Fig 6A). To further confirm the correct targeting of *CASP1* by CRISPR/Cas9, we also sequenced the genomic DNA spanning the CRISPR/Cas9 targeting region of the *CASP1*-sg1 and *CASP1*-sg2 cell lines. The sequencing results showed that frame shifts were introduced in 8 out of 13 clones for *CASP1*-sg1 and 12 out of 14 clones for *CASP1*-sg2 (S7 Fig). To evaluate the effects of caspase-1 depletion on EBV lytic reactivation, we triggered EBV reactivation by anti-IgG mediated BCR cross-linking. We found that the accumulation of the EBV lytic proteins ZTA and RTA was dramatically suppressed in the two *CASP1*-depleted cell lines upon BCR activation (Fig 6B). We also examined the level of lytic RNA transcripts in these cell lines. As expected, knockdown of *CASP1* dramatically suppressed the expression of immediate early (*ZTA* and *RTA*) and late (*BGLF2*) genes (Fig 6C). To test whether caspase-1 plays a role in EBV replication, we measured intracellular EBV genome copies following lytic induction. Compared with control, the intracellular viral DNA copies were significantly reduced upon *caspase-1* depletion (Fig 6D), suggesting that caspase-1 is required for EBV reactivation.

**Fig 7:**
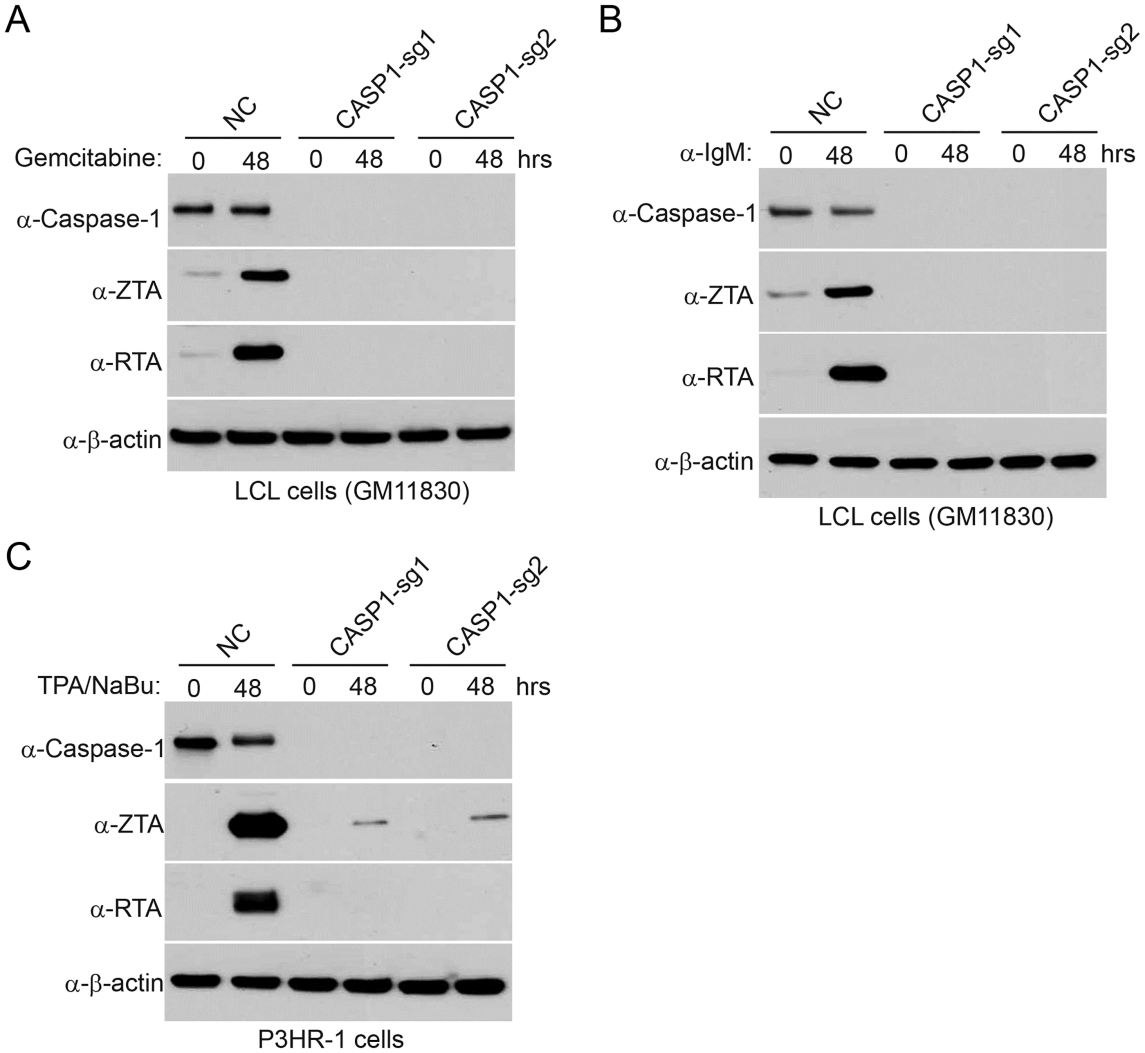
*CASP1* depletion suppresses EBV reactivation in LCL and P3HR-1 cells upon lytic induction. A and B. Control (NC) and *CASP1*-depleted (sg1 and sg2) LCL cells were either untreated (0 hr) or treated with 1 μg/mL gemcitabine (A) or 20 μg/mL α-IgM (B) for 48 hrs to induce lytic replication. Western blot analyses showing caspase-1, ZTA and RTA level as indicated. C. Control (NC) and *CASP1*-depleted (sg1 and sg2) P3HR-1 cells were either untreated (0 hr) or treated with TPA (20 ng/ml)/sodium butyrate (NaBu, 3 mM) for 48 hrs to induce lytic replication. Western blot analyses showing caspase-1, ZTA and RTA level as indicated.

To demonstrate the effect of caspase-1 in broader settings, we also depleted *CASP1* in P3HR-1 and EBV transformed LCL cells. We found that *CASP1*-depletion suppresses EBV reactivation treated with either gemcitabine, anti-IgM (for LCL) or TPA/sodium butyrate (for P3HR-1) (Fig 7), suggesting that IRF8/caspase-1 axis contributes to EBV reactivation upon lytic induction.

### Caspase-1 promotes EBV reactivation partially through KAP1 cleavage

**Fig 8:**
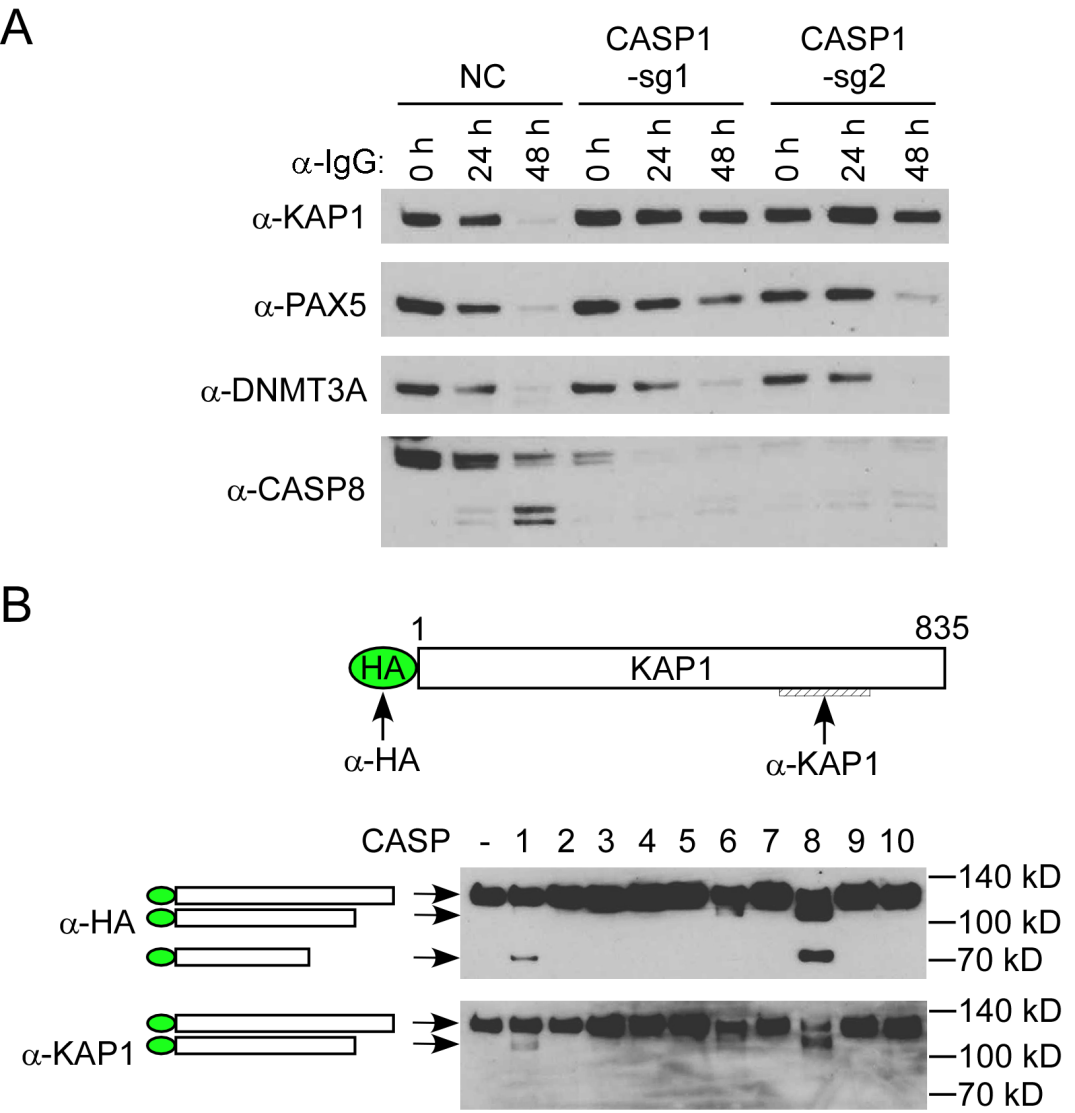
Caspase-1 promotes EBV reactivation partially through KAP1 cleavage. A. *Caspase-1* depletion suppresses KAP1 degradation. Protein extracts form Fig 5B were analyzed by western blot using antibodies against KAP1, PAX5, DNMT3A and Caspase-8 (CASP8). B. Caspase-1 and -8 cleave KAP1 *in vitro*. HA-KAP1 and the antibody recognition sites are labeled as indicated. HA-tagged KAP1 was immuoprecipitated from transfected 293T cells using HA magnetic beads. The beads-bound HA-KAP1 was incubated with individual recombinant caspase for 2 hrs at 37°C. WB was performed using either anti-HA or anti-KAP1 antibodies. The relative positions of cleaved fragments were labeled as indicated.

IRF8 can affect the degradation of KAP1, PAX5 and DNMT3A through caspase activation (Figs 3D and S3C). To test whether caspase-1 could affect their degradation, we monitored the protein stability when caspase-1 was depleted and lytic reactivation was induced by BCR activation. Interestingly, we found that the degradation of KAP1, but not PAX5 and DNMT3A, was blocked in caspase-1-depleted cells (Fig 8A). Based on these results, we reasoned that KAP1 might be cleaved by caspase-1. To prove this, we performed an *in vitro* cleavage assay using individual recombinant caspases and KAP1. To facilitate the detection of cleaved KAP1 fragments, we utilized an N-terminally HA-tagged KAP1 construct and immunoprecipitated the KAP1 protein from transfected 293T cells using HA magnetic beads. HA-KAP1 was eluted for the *in vitro* cleavage assay. Anti-HA and anti-KAP1 antibodies recognize N- and C-terminal of KAP1 respectively (Fig 8B), which facilitates the detection of cleaved fragments. Interestingly, we found that caspase-1, as well as caspase-8 can cleave KAP1 *in vitro* (Fig 8B). We also checked the expression of caspase-8 (CASP8) and found that the caspase-8 protein level (Fig 8A) but not its mRNA level (S8 Fig) was also reduced in caspase-1-depleted cells. These results together suggested that KAP1 cleavage is regulated by caspase-1 and -8 in Akata (EBV^+^) cells upon lytic induction. Because KAP1 depletion has been shown to facilitate EBV, Kaposi’s sarcoma-associated herpesvirus (KSHV) and human cytomegalovirus reactivation [12,57,58,59], we reasoned that the cleavage of KAP1 by caspase-1 and -8 should promote viral reactivation.

**Fig 9:**
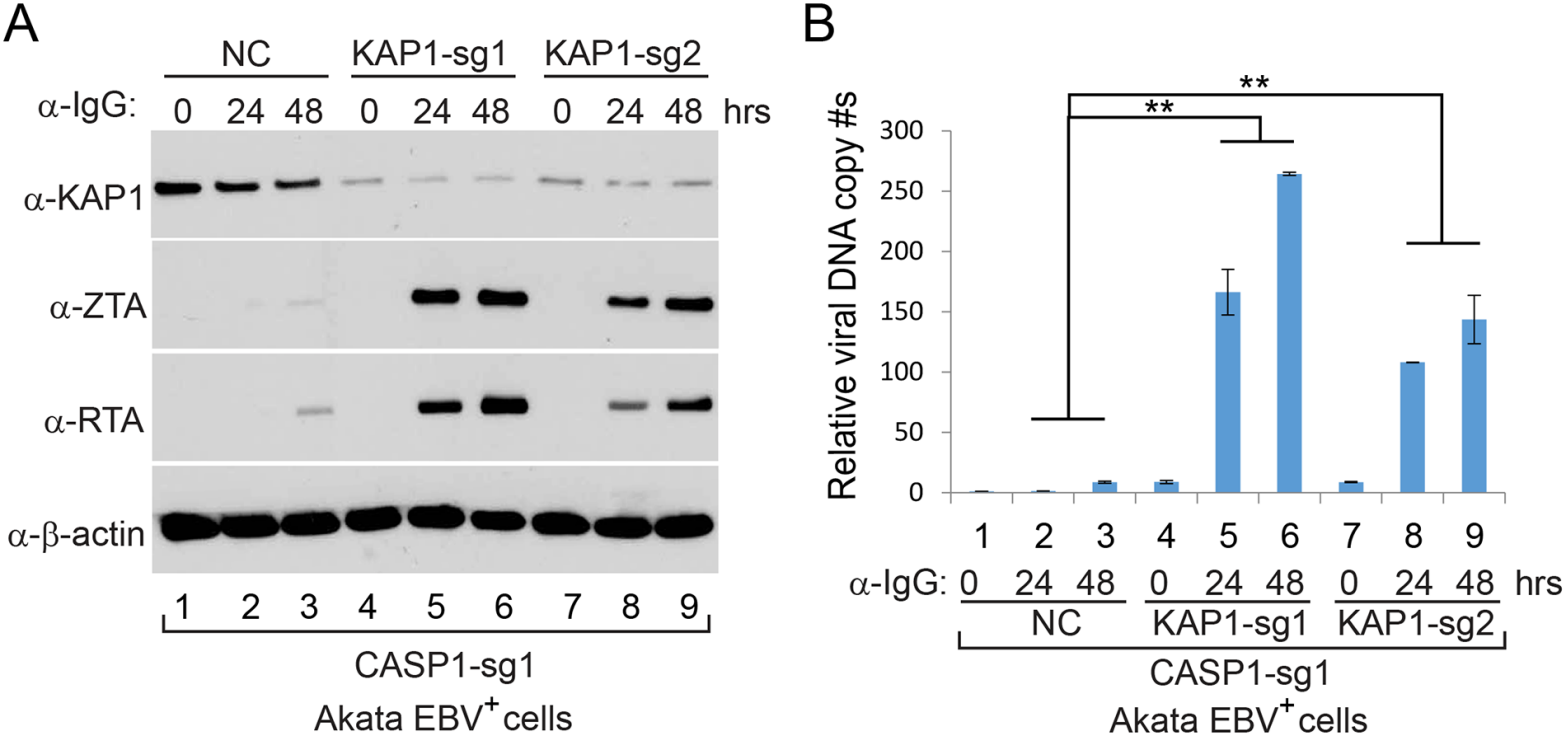
*KAP1* depletion facilitates EBV reactivation upon lytic induction. A. Control (NC) and *KAP1*-depleted (sg1 and sg2) Akata (EBV^+^)-*CASP1*-sg1 cells were untreated (0 hr) or treated with α-IgG (1:200) for 24 and 48 hrs to induce lytic replication. Western blot analyses showing KAP1, ZTA and RTA level as indicated. β-actin blot was included as loading controls. B. Intracellular viral DNA from cells treated as in (A) was measured by qPCR using primers to EBV *BALF5*. The value of NC control at 0 hr (lane 1) was set as 1. Data are presented as means ± standard deviations of triplicate assays. ** p<0.01.

To prove our prediction, we further depleted *KAP1* in *CASP1*-depleted (*sg1*) Akata cells by CRISPR/Cas9 genomic editing approach. As expected, *KAP1*-depletion in *CASP1*-depleted cells restored EBV reactivation upon BCR activation (Fig 9). Taken together, our results suggested that KAP1 is one of the important downstream targets of caspase-1 critical for EBV reactivation.

## Discussion

In this study, we discovered that the cellular factor IRF8 facilitates EBV lytic replication by promoting caspase expression and their activation upon lytic inducition. The IRF family proteins have been shown to play an important role in immunity, cell growth, differentiation and oncogenesis [19]. In contrast to the positive role of IRF8 in EBV lytic replication observed in our study, most of the IRFs contribute to anti-viral immunity and block the infection or lytic reactivation of herpesviruses. For example, it was reported that IRF1 restricts gammaherpesvirus replication through IFN-mediated suppression of viral replication [73,74,75,76]. IRF2 also suppresses gammaherpesvirus replication and reactivation by inhibiting the *M2* gene promoter [77]. Herpesviruses have evolved strategies to block IRF3 mediated anti-viral signaling [30,78]. IRF5 or IRF7-mediaed suppression of KSHV replication is counteracted by virally encoded proteins [79,80,81]. While IRF4 has been implicated in suppressing KSHV replication [82,83,84], it has been shown that IRF4 promotes gammaherpesvirus-68 replication through enhancing viral promoter activation [27,85,86]. Our identification of IRF8 as a positive regulator for EBV reactivation provides another example of IRFs in promoting herpesvirus lytic replication.

IRF8 is a unique member of the IRF family. It is highly expressed in B cells [87] and plays a critical role in B cell biology [88]. A recent study showed that IRF8 regulates EBV latency and the apoptosis of EBV-positive B cells [45]. However, the contribution of IRF8 to EBV lytic replication remained unclear prior to our study.

Using a CRISPR/Cas9 genomic editing method, we for the first time demonstrated that *IRF8* depletion dramatically suppresses the reactivation of EBV (Figs 1 and 4). IRF8 positively regulates apoptosis in different types of cells, including B cells [44,48,49,50,89]. Our RNA-seq and western blot analyses showed that IRF8 modulates caspase activation during EBV lytic replication (Figs 2 and 3). Especially, IRF8 binds to and enhances *CASP1* gene promoter activity (Fig 5) and caspase-1 expression is critical for EBV reactivation (Figs 6 and 7), partially through KAP1 cleavage (Figs 8 and 9). The regulation of caspase-1 by IRF8 may also contribute to subsequent BPLF1 cleavage, which has been shown to facilitate EBV DNA replication [90]. In addition, the cleavage of other cellular [56,91,92,93,94] or potentially viral proteins by caspase-1 and other caspases could also contribute to EBV reactivation. In addition to caspase cleavage of BPLF1, caspase-3 was reported cleave LMP1 in Hela cells while the functional importance is not clear [95]. Using bioinformatic tools PeptideCutter and GraBCas [96,97], we also predicted the potential caspase cleavage sites for EBV proteins and found that many other viral proteins, such as BCRF1/vIL10, may also be potentially cleaved by caspases (S2 Table). Further detailed studies are required to prove their cleavage and the subsequent functional importance during EBV reactivation.

Several studies showed that EBV lytic reactivation is closely associated with apoptosis and that caspase activation promotes EBV lytic replication in EBV-transformed LCLs and EBV-infected gastric cancer (AGS) cells [51,52]. However, the underlying mechanisms for caspase activation in EBV lytic replication were not clear. Here we provide evidence that caspase activation-induces de-stabilization of cellular factors KAP1, PAX5 and DNMT3A contributes to efficient EBV replication (Fig 3). KAP1 is a corepressor that inhibits the reactivation of multiple herpesviruses [12,57,58,98,99]. Although phosphorylation of KAP1 overcomes KAP1-mediated inhibition, our study suggested that caspase-1/-8-mediated cleavage provides another means to antagonize KAP1-mediated inhibition (Fig 8). PAX5 is a B-cell-specific transcription factor that promotes EBV latency and suppresses lytic reactivation [8,60,61,62]. A previous study suggested that the lytic triggers TPA and sodium butyrate facilitate PAX5 destabilization through down-regulation of its mRNA expression [8] and BCR stimulation of B cells also decreases the level of *PAX5* mRNA [100]. Our demonstration of caspase activation in PAX5 degradation provides an additional layer of regulation of PAX5 during EBV lytic replication. The *de novo* DNA methyltransferase DNMT3A contribute to γ-herpesvirus latency by suppressing viral lytic gene promoters through methylation [63]. It is conceivable that the down-regulation of DNMT3A by caspase activation would facilitate viral lytic replication.

Recent studies suggested that not only the decrease of IRF8 but also the increase of IRF4 is required for B cell differentiation, and that the IRF4/IRF8 ratios provide the differential signal for plasmablast versus germinal center plasma cell fate [70,88]. In Akata (EBV^+^) B cells, the expression of IRF4 is very low revealed by RNA-Seq (S6 Fig) and IRF4 protein is not detectable by western blot analysis [46]. Therefore, in the absence of IRF4, IRF8 depletion may not be sufficient to trigger B cell differentiation.

**Fig 10:**
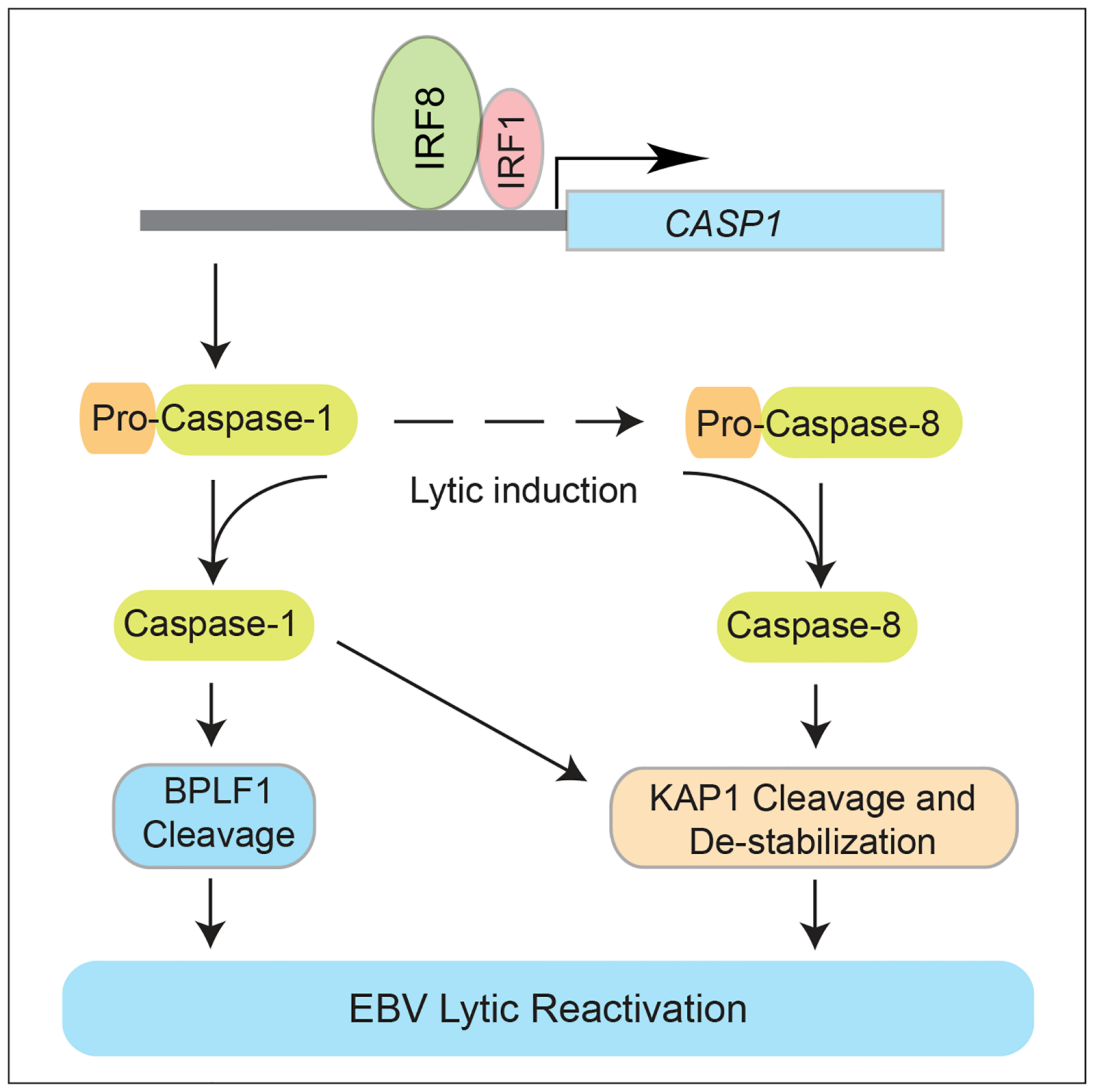
Hypothesized model by which IRF8 contributes to EBV lytic replication. IRF8 regulates the protein levels of caspase-1 and caspase-8. BCR stimulation triggers the activation of caspases and subsequent BPLF1 cleavage and the destabilization of KAP1, which leads to enhanced viral gene expression and DNA replication.

Although IRF8 normally suppresses B cell differentiation to plasma cells [101], a process that positively contributes to EBV reactivation [7], the results of our current work and the studies of others support a model in which IRF8 facilitates the reactivation of EBV upon lytic induction (Fig 10). IRF8 plays a key role in maintaining caspase-1 expression, a cellular protease critical for EBV reactivation upon lytic induction. Caspase-1 activation can trigger the specific cleavage of EBV BPLF1 for efficient viral DNA replication [90]. The activation of caspase-1 and caspase-8 can lead to the cleavage and destabilization of KAP1 and thus enhanced EBV replication.

As a positive regulator of interferon signaling, IRF8 might also function as an anti-viral factor [102,103,104] by promoting interferon signaling during primary EBV infection, which could then limit viral lytic infection and facilitate the establishment of latency. Future studies are required to examine this possibility.

In summary, our study suggests that IRF8 positively regulates EBV lytic replication upon lytic induction. These findings provide valuable insights into our understanding of IRF8 and caspase activation in EBV lytic replication, which lays the foundation for developing novel therapeutic strategies against EBV-associated malignancies.

## Materials and methods

### Cell culture and reagents

Akata (EBV^+^) cells (gifts from Diane Hayward, Johns Hopkins University) were grown in RPMI 1640 media supplemented with 10% FBS (Cat# 26140079, Thermo Fisher Scientific) in 5% CO_2_ at 37°C [105,106]. The P3HR-1 cell (ATCC, HTB-62) was purchased from ATCC. The EBV-transformed lymphoblast cell lines (LCL, GM11830) was purchased from the Coriell Institute for Medical Research (Camden, NJ). The P3HR-1 cell was grown in RPMI 1640 media supplemented with 10% FBS. The LCL cell was cultured in RPMI 1640 media supplemented with 15% FBS. 293T cells (a gift from Diane Hayward, Johns Hopkins University) were grown in DMEM media supplemented with 10% FBS. The pan-caspase inhibitor (Z-VAD-FMK, Cat# A1902) was purchased from ApexBio.

### Plasmids, cloning, and site-directed mutagenesis

Plasmid DNA was purified on miniprep columns according to the manufacturer’s protocol (Qiagen). pCMV3-N-FLAG and pCMV3-N-FLAG-IRF8 were obtained from Sino biological. pcDNA3.1-V5-His and pSG5 were obtained from Invitrogen and Stratagene, respectively. T vector pMD19 was bought from Clontech. pSG5-HA-KAP1 expression vector (pGL190) was a gift from Diane Hayward (Johns Hopkins) and contain the corresponding open reading frames in a derivative of pSG5 (Stratagene) [107]. The IRF1 ORF was cloned from Akata (EBV^+^) cDNA into pMD19 (Clontech) by PCR using the following primer sets: forward (5’-ATGCCCATCACTCGGATGC-3’) and reverse (5’-CTACGGTGCACAGGGAATGG-3’). IRF1 was then subcloned into pcDNA3.1-V5-His (Invitrogen) by using Gibson assembly and the following two primer sets: primer set-1, forward (5’-CCAGTGTGGTGGAATTGCCCTTGCTATGCCCATCACTCGGATGCGC-3’) and reverse (5’-CATTTTACCAACAGTACCGGAATGCCAAGCTTCGGTGCACAGGGAATGGCCTG-3’); primer set-2, forward (5’-CAGGCCATTCCCTGTGCACCGAAGCTTGGCATTCCGGTACTGTTGGTAAAATG-3’) and reverse (5’-GCGCATCCGAGTGATGGGCATAGCAAGGGCAATTCCACCACACTGG-3’). The pGL2-CASP1p1 (−488 to −8 relative to the *CASP1* ORF) and pGL2-CASP1p2 (−488 to -66) luciferase reporter plasmids were constructed into the pGL2-basic vector (Promega) by using the Gibson assembly and the following two primer sets for pGL2-CASP1p1: primer set-1, forward (5’-GCTCTTACGCGTGCTAGCTCGAGTGTGAAAAGAAGGACATTAAATAAGAA-3’) and reverse (5’-CAACAGTACCGGAATGCCAAGCTTCTCTCCTCCCTTCTTGTGTGAC-3’); primer set-2, forward (5’-GTCACACAAGAAGGGAGGAGAGAAGCTTGGCATTCCGGTACTGTTG-3’) and reverse (5’-TTCTTATTTAATGTCCTTCTTTTCACACTCGAGCTAGCACGCGTAAGAGC-3’); and the following two primer sets for pGL2-CASP1p2: primer set-1, forward (5’-GCTCTTACGCGTGCTAGCTCGAGTGTGAAAAGAAGGACATTAAATAAGAA-3’) and reverse (5’-CAACAGTACCGGAATGCCAAGCTTGGGCCTGTACATGTATTGGGAAATACTCAC-3’); primer set-2, forward (5’-GTGAGTATTTCCCAATACATGTACAGGCCCAAGCTTGGCATTCCGGTACTGTTG-3’) and reverse (5’-TTCTTATTTAATGTCCTTCTTTTCACACTCGAGCTAGCACGCGTAAGAGC-3’).

Plasmids pCMV3-N-FLAG-IRF8(K108E) and pGL2-CASP1p1-mut (IRF8 binding site mutation) were constructed by using QuikChange II site-directed mutagenesis kit (Agilent Technologies, Santa Clara, CA, USA) and the following primer sets: IRF8(K108E) forward (5’-GGACATTTCCGAGCCATACGAGGTTTACCGAATTGTTCCTG-3’) and reverse (5’-CAGGAACAATTCGGTAAACCTCGTATGGCTCGGAAATGTCC-3’); CASP1p1-mut forward (5’-CCAAAAAGGAAGGCGAAGCATACTTTCAGTGGAAGTCACACAAGAAGGGAGGAGAGAAGCTTG -3’) and reverse (5’-CAAGCTTCTCTCCTCCCTTCTTGTGTGACTTCCACTGAAAGTATGCTTCGCCTTCCTTTTTGG -3’) DNA sequences in all these plasmids were authenticated by automatic sequencing.

### *IRF8*, *CASP1* and *KAP1* depletion by CRISPR/Cas9 genomic editing

To deplete *IRF8* or *CASP1*, two different sgRNAs targeting human *IRF8* or *CASP1* were designed and cloned into lentiCRISPR v2 vector (a gift from Feng Zhang; Addgene plasmid # 52961) [108]. Packaging 293T cells were transfected with *IRF8* or *CASP1* sgRNAs or negative controls (non-targeting sgRNA-NC) and helper vectors (pMD2.G and psPAX2; gifts from Didier Trono; Addgene plasmid #s 12259 and 12260) using Lipofectamine 2000 reagent (Cat# 11668019, Life Technologies). Medium containing lentiviral particles and 8 μg/mL polybrene (Sigma-Aldrich, St. Louis) was used to infect Akata (EBV^+^) cells. Infected cells were selected in medium containing 2 μg/mL puromycin.

To deplete *KAP1*, two different sgRNAs targeting human *KAP1* were designed and cloned into lentiCRISPR v2-Blast vector (a gift from Mohan Babu, Addgene plasmid #83480). Packaging 293T cells were transfected with KAP1 sgRNAs or negative controls (non-targeting sgRNA-NC) and helper vectors (pMD2.G and psPAX2) using Lipofectamine 2000 reagent. Medium containing lentiviral particles and 8 μg/mL polybrene were used to infect caspase-1 knockout cell lines. Infected cells were selected in medium containing 10 μg/mL blasticidin.

The target guides sequences are as follows: *IRF8-sg1*: forward (5’-CACCGATTGACAGTAGCATGTATCC-3’) and reverse (5’-AAACGGATACATGCTACTGTCAATC-3’); *IRF8-sg2*: forward (5’-CACCGCGGAAATGTCCAGTTGGGAC-3’) and reverse (5’-AAACGTCCCAACTGGACATTTCCGC-3’); *CASP1-sg1*: forward (5’-CACCGGACAGTATTCCTAGAAGAAC-3’) and reverse (5’-AAACGTTCTTCTAGGAATACTGTCC-3’); *CASP1-sg2*: forward (5’-CACCGTTATCCGTTCCATGGGTGA-3’) and reverse (5’-AAACTCACCCATGGAACGGATAAC-3’); *sgRNA-NC*: forward (5’-CACCGTGAGGATCATGTCGAGCGCC-3’) and reverse (5’-AAACGGCGCTCGACATGATCCTCAC-3’); *KAP1-sg1*: forward (5’-CACCGGCGGGTGAAGTACACCAAGG-3’) and reverse (5’-AAACCCTTGGTGTACTTCACCCGCC-3’); *KAP1-sg2*: forward (5’-CACCGAGTCTCGGGATGGTGAACGT-3’) and reverse (5’-AAACACGTTCACCATCCCGAGACTC-3’).

### Sequencing of CRISPR targeting region

*IRF8* or *CASP1* knockdown efficiency was confirmed using western blot analysis and Sanger sequencing. In details, the PAM region (containing the target site of sgRNA) was amplified from DNA mixture extracted from three biological *IRF8-sg1*, *IRF8-sg2*, *CASP1-sg1* and *CASP1-sg2* pool cells, respectively by using Wizard Genomic DNA Purification Kit (Fisher). The primer sets used for cloning are as follows: *IRF8-sg1*: forward (5’-AATGGTGGTCGGCGGCTTC-3’) and reverse (5’-AATGGAGGCATCCACTTCCTGATT-3’); *IRF8-sg2*: forward (5’-GCCTGGGCAGTTTTTAAAGGGAAG-3’) and reverse (5’-TCGGTAAACTTTGTATGGCTCGGAAA-3’); *CASP1-sg1*: forward (5’-TCAATTCTGTTCCCCCTTTTCAAT-3’) and reverse (5’-AGGCTTGTGCTGCATGACTCTTAT-3’); *CASP1-sg2*: forward (5’-TGGGCTATTTCTGCTTCATTACTTT-3’) and reverse (5’-CCTTTCGGAATAACGGAGTCAATC-3’). The PCR amplicons were subcloned into pMD19 vectors (Clontech) and more than 10 clones were randomly chosen for sequencing.

### RNA-seq analysis

Total RNA from three biological replicates (cells derived from three distinct lentivrial transductions) was extracted using ISOLATE II RNA Mini Kit (Bioline). The library construction, cluster generation and HiSeq (Illumina) sequencing were performed with by the Genomics Sequencing Core of the Department of Environmental Health (University of Cincinnati) following the previous reported methods [109]. Raw fastq data were analyzed by using Galaxy (https://usegalaxy.org/). Human genome (hg38) was used as the reference genome. Differential gene expression between *IRF8*-depleted (*IRF8-sg2*) and control (NC) cells was analyzed by using DESeq2 [110]. The differentially expressed genes were selected based on a false-discovery rate–adjusted q-value (q< 0.05). Genes with more than 2-fold change were selected for further analysis. RNA-seq raw data have been submitted to National Center for Biotechnology Information (NCBI) Sequence Read Archive (SRA; accession numbers: SRP107862) with access URL https://www.ncbi.nlm.nih.gov/Traces/study/?acc=SRP107862.

### Chromatin-immunoprecipitation (ChIP)

2x10^7^ Akata (EBV^+^), LCL and P3HR-1 cells were cross-linked individually in 1% (w/v) formaldehyde (Sigma) for 5 min at room temperature and the cross-linking reaction was quenched by addition of glycine to a final concentration of 0.125M. Cells were washed twice with cold PBS and lysed in 1 ml of cell lysis buffer (10 mM Tris-HCl [pH 8.0], 10 mM NaCl, 0.2% [v/v] NP40, 10 mM Sodium butyrate, 50 μg/ml PMSF) with fresh added complete protease inhibitor on ice for 10 min. After centrifuge at 2,500 rpm at 4°C for 5 min, the supernatant was discarded and the nuclei were resuspended in 1.2 ml nuclei lysis buffer (50 mM Tris-HCl [pH 8.1], 10 mM EDTA, 1% [w/v] SDS, 10 mM Sodium butyrate, 50 μg/ml PMSF) with fresh added complete protease inhibitor on ice for 10 min. Then sonication was performed with a Diagenode Bioruptor 300. After extract clearing by centrifugation, supernatants were diluted 1:10 in dilution buffer (20 mM Tris-HCl [pH 8.1], 150 mM NaCl, 2 mM EDTA, 1% [v/v] Triton X-100, 0.01% [w/v] SDS, 10 mM Sodium butyrate 50 μg/ml PMSF) with fresh added complete protease inhibitor. Aliquots of each input chromatin lysate were reserved for PCR analysis. 1 ml of diluted chromatin lysate was incubated with ChIP-grade antibodies with rotation at 4°C overnight. Primary antibodies used were anti-IRF8 (Santa Cruz, Cat # sc-6058X), normal goat IgG (Santa Cruz, Cat # sc-2028), anti-IRF1 (abcam, Cat # ab26109), and normal rabbit IgG (Santa Cruz, Cat # sc-2027). 25 μl Protein A/G magnetic beads (life technologies, 10002D and 10004D) were added to each 1 ml ChIP and incubated for 2 hour at 4°C with rotation. Next, magnetic beads were pelleted with magnetic separation rack and washed once with cold low salt wash buffer (20 mM Tris-HCl [pH8.1], 2 mM EDTA, 150 mMNaCl, 1% [v/v] Triton X-100, 0.1% [w/v] SDS), once with high salt wash buffer (identical to low salt wash buffer, except 500 mM NaCl), once with LiCl wash buffer (10 mM Tris-HCl [pH8.1], 1 mM EDTA, 0.25 M LiCl, 1% [v/v] NP40, 1% Deoxycholic acid), and finally twice with TE buffer (10 mM Tris-HCl [pH8.1], 1 mM EDTA). Samples were then resuspended in 150 μl of elution buffer (0.1 M NaHCO3, 1% [w/v] SDS) and rotated for 20 min at room temperature. Two rounds of elution of protein-DNA complexes were pooled. Reversal of cross-linking was accomplished by incubation of pooled eluates at 65°C for 4 hours after addition of NaCl to final concentration of 200mM and 100 ug/ml Proteinase K. DNA was purified by phenol-chloroform extraction followed by isopropanol-sodium acetate precipitation and then resuspended in 100 μl nuclease-free water and quantified using regular PCR. Purified input chromatin lysate was used in PCR reactions for standardization. ChIP primers used to amplify the *CASP1* promoter are: forward (5’-TACACTACCTGATGCAGGCTA-3’) and reverse (5’-TGAAACTGAAAGTATGCTTCG-3’).

### Reverse transcription and quantitative PCR (RT-qPCR)

Total RNA was extracted using ISOLATE II RNA Mini Kit (Bioline). Reverse transcription was carried out by using High Capacity cDNA Reverse Transcription Kit (Invitrogen). Quantitative PCR (qPCR) was performed using an ABI Prism 7000 Sequence Detector with SYBR Green. The PCR reactions were set up in a 96-well optical plate in duplicate by adding the following reagents into each well: 2 μl of cDNA, 10 μl of SYBR Green PCR Master Mix (Applied Biosystems, Foster City, CA, USA); the final concentrations of primers were 0.3 μmol/L in a final volume of 20 μl. The PCR amplification protocol was initiated at 50°C for 2 min followed by 10 min at 95°C and 40 PCR cycles consisting of 15 seconds at 95°C followed by 60°C for 1 min. All samples were tested with the reference gene *β-actin* for data normalization to correct for variations in RNA quality and quantity. The specificity of amplification of targets with high Ct values was confirmed by analysis of the temperature dissociation curves. Primers used for measuring gene transcriptional level: *RTA* and *β-actin* primers were described previously [13]; *ZTA* primers are forward 5’-AGGCCAGCTCACTGCCTATC-3’ and reverse 5’-TGATTCTGGGTTATGTCTGA-3’; *BGLF2* primers are forward 5’-ATCTGGCACCTGTCCTTGTC-3’ and reverse5’-GGGACCTCTTTCCCATTAGC-3’; *BGLF4* primers are forward 5’-GGCAATAGAGGCGATAGAGC-3’ and reverse 5’-TGGTCCTGACTGATTATGGG-3’; *CASP1* primers are forward 5’-ATAGCTGGGTTGTCCTGCAC-3’ and reverse 5’-GCCAAATTTGCATCACATACA-3’; *AIM2* primers are forward 5’-TAGCGCCTCACGTGTGTTAG-3’ and reverse 5’-TTGAAGCGTGTTGATCTTCG-3’; *IFNB1* primers are forward 5’-CAGGAGAGCAATTTGGAGGA-3’ and reverse 5’-CTTTCGAAGCCTTTGCTCTG-3’; *SLAMF7* primers are forward 5’-GAACCGACCAGCTCTTTCAC-3’ and reverse 5’-AATATGGCTGGTTCCCCAAC-3’; *SULF1* primers are forward 5’-ATCCTGGTTGAATAATCAATCTCT-3’ and reverse 5’-ATGCAGGTTCTTCAAGGCAG-3’; *TNFSF10* primers are forward 5’-AGCAATGCCACTTTTGGAGT-3’ and reverse 5’-TTCACAGTGCTCCTGCAGTC-3’; *MX1* primers are forward 5’-GATGATCAAAGGGATGTGGC-3’ and reverse 5’-AGCTCGGCAACAGACTCTTC-3’; *DAPL1* primers are forward 5’-TGCCCTGAATGACGCACTG-3’ and reverse 5’-GTGGGTTTTTGATGCGCCAT-3’; CASP8 primers are forward 5’-TGTCCAGTTGTTCCCCAATA-3’ and reverse 5’-GGTCACTTGAACCTTGGGAA-3’.

### IRF8 reconstitution

The pLX304 vector was a gift from David Root (Addgene plasmid # 25890). The V5-tagged pLX304-IRF8 was purchased from DNASU Plasmid Repository. To prepare lentiviruses, 293T cells were transfected with empty vector or pLX304 containing the gene of *IRF8* and the help vectors (pMD2.G and psPAX2) using Lipofectamine 2000 reagent. The supernatants were harvested at 48 h after transfection. The medium containing lentiviral particles and 8 μg/mL polybrene were used to infect *IRF8*-depleted (sg2) cell lines. Infected cells were selected in medium containing 10 μg/mL blasticidin.

### Luciferase reporter assay

Luciferase assay was performed as previously described [16]. Briefly, 293T cells were co-transfected with the firefly luciferase reporter vectors along with IRF8 (WT or K108E mutant), IRF1, and renilla expression plasmids using Lipofectamine 2000 reagent (Cat# 11668019, Life Technologies). The Akata (EBV^+^) cell was transfected using electroporation method. For plasmid transfection, 10 μg each of plasmid were mixed with 5x10^6^ cells in a 4-mm cuvette. Electroporation was performed at 970 μF and 0.2 V with a Gene pulser Xcell system (Bio-Rad). The cells were transferred to new plates contain 10 ml pre-warmed fresh medium. At thirty-six hours post-transfection, cell extracts were prepared and assayed with the dual-luciferase assay kit from Promega (Cat #E1960, Madison, WI, USA). Each condition was performed in triplicate.

### Lytic induction and measurement of viral DNA copy number

Akata (EBV^+^) cells were treated with 50 μg/ml of goat anti-human IgG (MP Biomedicals) for 24 and 48 h to induce the EBV lytic cycle. For caspase inhibition assay, Akata (EBV^+^) cells were untreated or pretreated with pan-caspase inhibitor for 1 hr and then treated with anti-IgG (1:200, Cat# 55087, MP Biomedicals) for additional 48 hrs. EBV reactivation in P3HR-1 cells was triggered by addition of TPA (20 ng/ml) and sodium butyrate (3 mM; Millipore, Cat# 19–137). The EBV lytic replication in LCL cells was induced by addition of gemcitabine (1 μg/mL; Fisher Scientific, Cat# NC9325685). To induce the BCR activation, the LCL cells were treated with anti-IgM antibody (20 μg/mL, Cat# 2020–01, Southern Biotech) for 0 to 48 hrs.

To measure EBV replication, intracellular viral DNA and virion-associated DNA present in culture supernatant were determined by qPCR analysis [13]. Total genomic DNA was extracted by using Wizard Genomic DNA Purification Kit (Promega, Madison, WI, USA). For extracellular viral DNA extraction, the supernatant (120 μl) was treated with 4 μl RQ1 DNase (Promega) for 1 h at 37°C, and reactions were stopped by adding 20 μl of stop buffer and incubation at 65°C for 10 min; 12.5 μl proteinase K (20 mg/ml, Invitrogen) and 25 μl 10% (wt/vol) SDS then were added to the reaction mixtures, which were incubated for 1 h at 65°C. DNA was purified by phenol-chloroform extraction followed by isopropanol-sodium acetate precipitation and then resuspended in 100 μl nuclease-free water. qPCR was performed as mentioned above. Relative levels of viral DNA were normalized to supernatant viral DNA without lytic induction. The *BALF5* primers used for quantitating EBV copy numbers were described previously [13,105]. The reference gene *β-actin* was used for data normalization.

### *In vitro* caspase cleavage assay

*In vitro* cleavage assay was performed as previously described [94]. Briefly, HA-tagged KAP1 was immunoprecipitated from transfected 293T cells using HA magnetic beads. The beads-bound HA-KAP1 and individual active caspases (active human caspases group IV; ApexBio, Cat# K2060) were incubated in caspase assay buffer (50 mM HEPES, pH7.2, 50 mM NaCl, 0.1% Chaps, 10 mM EDTA, 5% Glycerol and 10mM DTT) at 37°C for 2 hrs. Reactions were stopped by boiling in 2× SDS sample buffer and samples were analyzed by western blot.

### Immunoblot analysis

Cell lysates were harvested in lysis buffer including protease inhibitors (Roche) as described previously[106]. Protein concentration was determined using the Bradford assay (Biorad), and proteins were separated in SDS 4–20% polyacrylamide gels and then transferred onto a PVDF membrane. Membranes were blocked in TBS containing 5% milk, and 0.1% Tween 20 solution. Membranes were then incubated in the following primary antibodies: mouse anti-ZTA (Argene, Cat # 11–007, 1:5,000), mouse anti-RTA (Argene, 1:1,000), mouse anti-BGLF4 antibody (1:1,000) [111], anti-β-actin (Sigma, Cat # A5441, 1:5,000), anti-IRF8 (CST, Cat #5628, 1:1,000), anti-PARP (CST, Cat #9532, 1:1,000), anti-Cleaved PARP (CST, Cat #5625, 1:1,000), anti-Cleaved Caspase Substrates (CST, Cat #8698, 1:1,000), anti-Caspase-1 (CST, Cat #3866, 1:1,000), anti-Caspase-2 (CST, Cat #2224, 1:1,000), anti-Caspase-3 (Santa Cruz, Cat #sc-7148, 1:1,000), anti-Cleaved Caspase-3 (CST, Cat #9664, 1:1,000), anti-Caspase-7 (CST, Cat #12827, 1:1,000), anti-Cleaved Caspase-7 (CST, Cat #8438, 1:1,000), anti-Caspase-8 (CST, Cat #9746, 1:1,000), anti-Cleaved Caspase-8 (CST, Cat #9496, 1:1,000), anti-Caspase-9 (CST, Cat #9508, 1:1,000), anti-Bcl-2 (Bethyl, Cat #A303-675A, 1:1,000), anti-KAP1 (CST, Cat #4123, 1:1,000), anti-PAX5 (CST, Cat #8970, 1:1,000), anti-DNMT3A (Bethyl, Cat #A304-278A, 1:1,000), anti-STAT3 (CST, Cat #9139, 1:1,000), and anti-HA (CST, Cat #14031S, 1:1,000). The secondary antibodies used were horseradish peroxidase (HRP)-labeled goat anti-mouse antibody (Fisher Scientific, 1:5,000) and HRP-labeled anti-rabbit antibody (Fisher scientific, 1:5,000).

### Bioinformatics analysis

Potential caspase cleavage sites were searched for all the EBV protein sequences using PeptideCutter (http://web.expasy.org/peptide_cutter/) and the GraBCas software [96,97].

### Statistical analysis

All numerical data were presented as mean ± standard deviation of triplicate assays. The statistical significances were determined using Student’s two-tail t-test, where p<0.05 was considered statistically significant.

## Supporting information

S1 Table A. The transcription level of all the genes identified in *IRF8-sg2* and NC cell lines. B. Differentially expressed genes between *IRF8-sg2* and NC cell lines.
(XLSX)

S2 Table The predicted caspase cleavage sites on EBV proteins. (XLSX)

S1 Fig *IRF8* depletion efficiency evaluated by Sanger sequencing. The sequencing of *IRF8*-depleted cell lines showing that 10 out of 22 clones for sg1 and 9 out of 14 clones for sg2 contain frame shifts. The PAM sequences were highlighted by red and the guide RNA sequences were shown in bold. WT: wild-type; “+” or “-” followed by numbers indicates the number of base pair inserted or deleted; “S” followed by numbers indicates the number of site mutations; “×” followed by numbers indicates the number of clones obtained in the sequencing.
(TIF)

S2 Fig *IRF8* reconstitution facilitates EBV lytic replication upon lytic induction. A. Akata (EBV^+^) *IRF8*-sg2 cells were used to establish *IRF8*-expressing stable cell lines using a pLX-*IRF8* lentiviral construct. Western blot analyses showing IRF8, ZTA, RTA and BGLF4 expression level in different cell lines upon IgG cross-linking as indicated. B. Intracellular viral DNA from cells treated as in (A) was measured by qPCR using primers to EBV *BALF5*. The value of vector control at 0 hr (lane 4) was set as 1. Data are presented as means ± standard deviations of triplicate assays. ** p<0.01.
(TIF)

S3 Fig *IRF8* depletion suppresses caspase activation. A. RT-qPCR validation of the 8 apoptosis-related genes in *IRF8-sg1* cells. B and C. *IRF8* depletion (*sg1*) suppresses caspase-1 expression and the generation of cleaved caspase substrates upon lytic induction by anti-IgG cross-linking. Western blot analysis of protein extracts from Fig 1C using antibodies against caspase-1, PARP, and cleaved caspase substrates as indicated in panel (B). Western blot analysis of protein extracts from Fig 1C using antibodies against caspase-3, cleaved caspase-3, caspase-8, cleaved caspase-8, caspase-7, cleaved caspase-7, caspase-9, cleaved caspass-9, caspase-2, Bcl2, KAP1, PAX5, DNMT3A and STAT3 as indicated in panel (C).
(TIF)

S4 Fig The relative expression level of *CASPs* in the control (NC) or *IRF8*-depleted (sg2) Akata (EBV^+^) cells obtained by RNA-seq analysis. RPKM, Reads Per Kilobase of transcript per Million mapped reads.
(TIF)

S5 Fig IRF8 and IRF1 bind to *CASP1* promoter and activate the promoter activity in B cells. A. ChIP-PCR analysis using three EBV-positive cells [Akata (EBV^+^), P3HR-1 and LCLs] showing IRF8/IRF1 binding to *CASP1* promoter. ChIP by a nonspecific IgG was include as negative controls. B. The pGL2-*CASP1p-1-Luc* constructs were co-transfected into Akata (EBV^+^) cells with either 10 ug of IRF8, IRF1 or IRF8-K108E expression vectors. Luciferase assays were performed 36 hrs post-transfection. The value of cells transfected with an empty vector was set as 1. The results were presented as mean ± standard deviation of triplicate assays. ** p<0.01, *** p<0.001.
(TIF)

S6 Fig The relative expression level of *IRFs* in the control (NC) Akata (EBV^+^) cells obtained by RNA-seq analysis. RPKM, Reads Per Kilobase of transcript per Million mapped reads.
(TIF)

S7 Fig *CASP1* depletion efficiency evaluated by Sanger sequencing. The sequencing of *CASP1*-depleted cell lines showing that 8 out of 13 clones for *CASP1*-sg1 and 12 out of 14 clones for *CASP1*-sg2 contain frame shifts. The PAM sequences were highlighted by red and the guide RNA sequences were shown in bold. WT: wild-type; “+” or “-” followed by numbers indicates the number of base pair inserted or deleted; “S” followed by numbers indicates the number of site mutations; “×” followed by numbers indicates the number of clones obtained in the sequencing.
(TIF)

S8 Fig *CASP8* mRNA level upon *CASP1* depletion. qPCR analysis showing that *CASP8* mRNA level was slightly increased by *CASP1* depletion. The value was normalized by qPCR using specific primers to *β-actin*. Data are presented as means ± standard deviations of triplicate assays.
(TIF)

## References

[1] Young LS, Yap LF, Murray PG (2016) Epstein-Barr virus: more than 50 years old and still providing surprises. Nature Reviews Cancer 16: 789–802. doi: 10.1038/nrc.2016.92 27687982 10.1038/nrc.2016.92

[2] Hammerschmidt W, Sugden B (2013) Replication of Epstein–Barr Viral DNA. Cold Spring Harbor perspectives in biology 5: a013029 doi: 10.1101/cshperspect.a013029 23284049 10.1101/cshperspect.a013029 PMC3579399

[3] Zalani S, Holley-Guthrie E, Kenney S (1995) The Zif268 cellular transcription factor activates expression of the Epstein-Barr virus immediate-early BRLF1 promoter. J Virol 69: 3816–3823. 7745729 10.1128/jvi.69.6.3816-3823.1995 PMC189099

[4] Wu FY, Wang SE, Chen H, Wang L, Hayward SD, et al (2004) CCAAT/enhancer binding protein alpha binds to the Epstein-Barr virus (EBV) ZTA protein through oligomeric interactions and contributes to cooperative transcriptional activation of the ZTA promoter through direct binding to the ZII and ZIIIB motifs during induction of the EBV lytic cycle. J Virol 78: 4847–4865. doi: 10.1128/JVI.78.9.4847-4865.2004 15078966 10.1128/JVI.78.9.4847-4865.2004 PMC387681

[5] Robinson AR, Kwek SS, Kenney SC (2012) The B-cell specific transcription factor, Oct-2, promotes Epstein-Barr virus latency by inhibiting the viral immediate-early protein, BZLF1. PLoS Pathog 8: e1002516 doi: 10.1371/journal.ppat.1002516 22346751 10.1371/journal.ppat.1002516 PMC3276558

[6] Robinson AR, Kwek SS, Hagemeier SR, Wille CK, Kenney SC (2011) Cellular transcription factor Oct-1 interacts with the Epstein-Barr virus BRLF1 protein to promote disruption of viral latency. J Virol 85: 8940–8953. doi: 10.1128/JVI.00569-11 21697476 10.1128/JVI.00569-11 PMC3165789

[7] Reusch JA, Nawandar DM, Wright KL, Kenney SC, Mertz JE (2015) Cellular differentiation regulator BLIMP1 induces Epstein-Barr virus lytic reactivation in epithelial and B cells by activating transcription from both the R and Z promoters. J Virol 89: 1731–1743. doi: 10.1128/JVI.02781-14 25410866 10.1128/JVI.02781-14 PMC4300755

[8] Raver RM, Panfil AR, Hagemeier SR, Kenney SC (2013) The B-cell-specific transcription factor and master regulator Pax5 promotes Epstein-Barr virus latency by negatively regulating the viral immediate early protein BZLF1. J Virol 87: 8053–8063. doi: 10.1128/JVI.00546-13 23678172 10.1128/JVI.00546-13 PMC3700198

[9] Nawandar DM, Wang A, Makielski K, Lee D, Ma S, et al (2015) Differentiation-Dependent KLF4 Expression Promotes Lytic Epstein-Barr Virus Infection in Epithelial Cells. PLoS Pathog 11: e1005195 doi: 10.1371/journal.ppat.1005195 26431332 10.1371/journal.ppat.1005195 PMC4592227

[10] Nawandar DM, Ohashi M, Djavadian R, Barlow E, Makielski K, et al (2017) Differentiation-Dependent LMP1 Expression Is Required for Efficient Lytic Epstein-Barr Virus Reactivation in Epithelial Cells. J Virol 91. 10.1128/JVI.02438-16 PMC5375685 28179525

[11] Murata T, Narita Y, Sugimoto A, Kawashima D, Kanda T, et al (2013) Contribution of myocyte enhancer factor 2 family transcription factors to BZLF1 expression in Epstein-Barr virus reactivation from latency. J Virol 87: 10148–10162. doi: 10.1128/JVI.01002-13 23843637 10.1128/JVI.01002-13 PMC3754021

[12] Li X, Burton EM, Bhaduri-McIntosh S (2017) Chloroquine triggers Epstein-Barr virus replication through phosphorylation of KAP1/TRIM28 in Burkitt lymphoma cells. PLoS Pathog 13: e1006249 doi: 10.1371/journal.ppat.1006249 28249048 10.1371/journal.ppat.1006249 PMC5348047

[13] Li R, Zhu J, Xie Z, Liao G, Liu J, et al (2011) Conserved herpesvirus kinases target the DNA damage response pathway and TIP60 histone acetyltransferase to promote virus replication. Cell Host Microbe 10: 390–400. doi: 10.1016/j.chom.2011.08.013 22018239 10.1016/j.chom.2011.08.013 PMC3253558

[14] Kraus RJ, Perrigoue JG, Mertz JE (2003) ZEB negatively regulates the lytic-switch BZLF1 gene promoter of Epstein-Barr virus. J Virol 77: 199–207. doi: 10.1128/JVI.77.1.199-207.2003 12477825 10.1128/JVI.77.1.199-207.2003 PMC140584

[15] Iempridee T, Reusch JA, Riching A, Johannsen EC, Dovat S, et al (2014) Epstein-Barr virus utilizes Ikaros in regulating its latent-lytic switch in B cells. J Virol 88: 4811–4827. doi: 10.1128/JVI.03706-13 24522918 10.1128/JVI.03706-13 PMC3993812

[16] Huang J, Liao G, Chen H, Wu FY, Hutt-Fletcher L, et al (2006) Contribution of C/EBP proteins to Epstein-Barr virus lytic gene expression and replication in epithelial cells. J Virol 80: 1098–1109. doi: 10.1128/JVI.80.3.1098-1109.2006 16414987 10.1128/JVI.80.3.1098-1109.2006 PMC1346937

[17] Kosowicz JG, Lee J, Peiffer B, Guo Z, Chen J, et al (2017) Drug Modulators of B Cell Signaling Pathways and Epstein-Barr Virus Lytic Activation. J Virol 91. 10.1128/JVI.00747-17 PMC5533897 28566383

[18] Takada K (1984) Cross-linking of cell surface immunoglobulins induces Epstein-Barr virus in Burkitt lymphoma lines. Int J Cancer 33: 27–32. 6319296 10.1002/ijc.2910330106

[19] Tamura T, Yanai H, Savitsky D, Taniguchi T (2008) The IRF family transcription factors in immunity and oncogenesis. Annu Rev Immunol 26: 535–584. doi: 10.1146/annurev.immunol.26.021607.090400 18303999 10.1146/annurev.immunol.26.021607.090400

[20] Mamane Y, Heylbroeck C, Génin P, Algarté M, Servant MJ, et al (1999) Interferon regulatory factors: the next generation. Gene 237: 1–14. 10524230 10.1016/s0378-1119(99)00262-0

[21] Savitsky D, Tamura T, Yanai H, Taniguchi T (2010) Regulation of immunity and oncogenesis by the IRF transcription factor family. Cancer immunology, immunotherapy 59: 489–510. doi: 10.1007/s00262-009-0804-6 20049431 10.1007/s00262-009-0804-6 PMC11030943

[22] Schaefer BC, Paulson E, Strominger JL, Speck SH (1997) Constitutive activation of Epstein-Barr virus (EBV) nuclear antigen 1 gene transcription by IRF1 and IRF2 during restricted EBV latency. Molecular and cellular biology 17: 873–886. 9001242 10.1128/mcb.17.2.873 PMC231814

[23] Ning S, Huye LE, Pagano JS (2005) Interferon regulatory factor 5 represses expression of the Epstein-Barr virus oncoprotein LMP1: braking of the IRF7/LMP1 regulatory circuit. Journal of virology 79: 11671–11676. doi: 10.1128/JVI.79.18.11671-11676.2005 16140744 10.1128/JVI.79.18.11671-11676.2005 PMC1212628

[24] Xu D, Meyer F, Ehlers E, Blasnitz L, Zhang L (2011) Interferon regulatory factor 4 (IRF-4) targets IRF-5 to regulate Epstein-Barr virus transformation. Journal of Biological Chemistry 286: 18261–18267. doi: 10.1074/jbc.M110.210542 21454650 10.1074/jbc.M110.210542 PMC3093898

[25] Xu D, Zhao L, Del Valle L, Miklossy J, Zhang L (2008) Interferon regulatory factor 4 is involved in Epstein-Barr virus-mediated transformation of human B lymphocytes. Journal of virology 82: 6251–6258. doi: 10.1128/JVI.00163-08 18417578 10.1128/JVI.00163-08 PMC2447047

[26] Zhang L, Pagano JS (1999) Interferon regulatory factor 2 represses the Epstein-Barr virus BamHI Q latency promoter in type III latency. Molecular and cellular biology 19: 3216–3223. 10082588 10.1128/mcb.19.4.3216 PMC84115

[27] O’Flaherty BM, Soni T, Wakeman BS, Speck SH (2014) The murine gammaherpesvirus immediate-early Rta synergizes with IRF4, targeting expression of the viral M1 superantigen to plasma cells. PLoS Pathog 10: e1004302 doi: 10.1371/journal.ppat.1004302 25101696 10.1371/journal.ppat.1004302 PMC4125235

[28] Bentz GL, Liu R, Hahn AM, Shackelford J, Pagano JS (2010) Epstein–Barr virus BRLF1 inhibits transcription of IRF3 and IRF7 and suppresses induction of interferon-β. Virology 402: 121–128. doi: 10.1016/j.virol.2010.03.014 20381110 10.1016/j.virol.2010.03.014 PMC2871977

[29] Hahn AM, Huye LE, Ning S, Webster-Cyriaque J, Pagano JS (2005) Interferon regulatory factor 7 is negatively regulated by the Epstein-Barr virus immediate-early gene, BZLF-1. Journal of virology 79: 10040–10052. doi: 10.1128/JVI.79.15.10040-10052.2005 16014964 10.1128/JVI.79.15.10040-10052.2005 PMC1181586

[30] Wang J-T, Doong S-L, Teng S-C, Lee C-P, Tsai C-H, et al (2009) Epstein-Barr virus BGLF4 kinase suppresses the interferon regulatory factor 3 signaling pathway. Journal of virology 83: 1856–1869. doi: 10.1128/JVI.01099-08 19052084 10.1128/JVI.01099-08 PMC2643756

[31] Lee CH, Melchers M, Wang H, Torrey TA, Slota R, et al (2006) Regulation of the germinal center gene program by interferon (IFN) regulatory factor 8/IFN consensus sequence-binding protein. The Journal of experimental medicine 203: 63–72. doi: 10.1084/jem.20051450 16380510 10.1084/jem.20051450 PMC2118063

[32] Huang W, Horvath E, Eklund EA (2007) PU. 1, interferon regulatory factor (IRF) 2, and the interferon consensus sequence-binding protein (ICSBP/IRF8) cooperate to activate NF1 transcription in differentiating myeloid cells. Journal of Biological Chemistry 282: 6629–6643. doi: 10.1074/jbc.M607760200 17200120 10.1074/jbc.M607760200

[33] Huang W, Zhu C, Wang H, Horvath E, Eklund EA (2008) The interferon consensus sequence-binding protein (ICSBP/IRF8) represses PTPN13 gene transcription in differentiating myeloid cells. Journal of Biological Chemistry 283: 7921–7935. doi: 10.1074/jbc.M706710200 18195016 10.1074/jbc.M706710200

[34] Kautz B, Kakar R, David E, Eklund EA (2001) SHP1 Protein-tyrosine Phosphatase Inhibits gp91PHOXand p67PHOX Expression by Inhibiting Interaction of PU. 1, IRF1, Interferon Consensus Sequence-binding Protein, and CREB-binding Protein with Homologous Cis Elements in the CYBB andNCF2 Genes. Journal of Biological Chemistry 276: 37868–37878. doi: 10.1074/jbc.M103381200 11483597 10.1074/jbc.M103381200

[35] Unlu S, Kumar A, Waterman WR, Tsukada J, Wang KZ, et al (2007) Phosphorylation of IRF8 in a pre-associated complex with Spi-1/PU. 1 and non-phosphorylated Stat1 is critical for LPS induction of the IL1B gene. Molecular immunology 44: 3364–3379. doi: 10.1016/j.molimm.2007.02.016 17386941 10.1016/j.molimm.2007.02.016 PMC2719065

[36] Chang T-H, Xu S, Tailor P, Kanno T, Ozato K (2012) The small ubiquitin-like modifier-deconjugating enzyme sentrin-specific peptidase 1 switches IFN regulatory factor 8 from a repressor to an activator during macrophage activation. The Journal of Immunology 189: 3548–3556. doi: 10.4049/jimmunol.1201104 22942423 10.4049/jimmunol.1201104 PMC4158928

[37] Kim JY, Ozato K (2009) The sequestosome 1/p62 attenuates cytokine gene expression in activated macrophages by inhibiting IFN regulatory factor 8 and TNF receptor-associated factor 6/NF-κB activity. The Journal of Immunology 182: 2131–2140. doi: 10.4049/jimmunol.0802755 19201866 10.4049/jimmunol.0802755 PMC4151355

[38] Kong HJ, Anderson DE, Lee CH, Jang MK, Tamura T, et al (2007) Cutting edge: autoantigen Ro52 is an interferon inducible E3 ligase that ubiquitinates IRF-8 and enhances cytokine expression in macrophages. The Journal of Immunology 179: 26–30. 17579016 10.4049/jimmunol.179.1.26

[39] Tamura T, Nagamura-Inoue T, Shmeltzer Z, Kuwata T, Ozato K (2000) ICSBP directs bipotential myeloid progenitor cells to differentiate into mature macrophages. Immunity 13: 155–165. 10981959 10.1016/s1074-7613(00)00016-9

[40] Tsujimura H, Tamura T, Gongora C, Aliberti J, e Sousa CR, et al (2003) ICSBP/IRF-8 retrovirus transduction rescues dendritic cell development in vitro. Blood 101: 961–969. doi: 10.1182/blood-2002-05-1327 12393459 10.1182/blood-2002-05-1327

[41] White CL, Kessler PM, Dickerman BK, Ozato K, Sen GC (2016) Interferon Regulatory Factor 8 (IRF8) Impairs Induction of Interferon Induced with Tetratricopeptide Repeat Motif (IFIT) Gene Family Members. Journal of Biological Chemistry 291: 13535–13545. doi: 10.1074/jbc.M115.705467 27137933 10.1074/jbc.M115.705467 PMC4919440

[42] Fragale A, Stellacci E, Ilari R, Remoli AL, Lanciotti A, et al (2011) Critical role of IRF-8 in negative regulation of TLR3 expression by Src homology 2 domain-containing protein tyrosine phosphatase-2 activity in human myeloid dendritic cells. The Journal of Immunology 186: 1951–1962. doi: 10.4049/jimmunol.1000918 21220691 10.4049/jimmunol.1000918 PMC4053178

[43] Xu Y, Jiang L, Fang J, Fang R, Morse HC III, et al (2015) Loss of IRF8 Inhibits the Growth of Diffuse Large B-cell Lymphoma. Journal of Cancer 6: 953 doi: 10.7150/jca.12067 26316891 10.7150/jca.12067 PMC4543755

[44] Gabriele L, Phung J, Fukumoto J, Segal D, Wang IM, et al (1999) Regulation of apoptosis in myeloid cells by interferon consensus sequence-binding protein. J Exp Med 190: 411–421. 10430629 10.1084/jem.190.3.411 PMC2195590

[45] Banerjee S, Lu J, Cai Q, Saha A, Jha HC, et al (2013) The EBV latent antigen 3C inhibits apoptosis through targeted regulation of interferon regulatory factors 4 and 8. PLoS Pathog 9: e1003314 doi: 10.1371/journal.ppat.1003314 23658517 10.1371/journal.ppat.1003314 PMC3642079

[46] Wang L, Yao ZQ, Moorman JP, Xu Y, Ning S (2014) Gene expression profiling identifies IRF4-associated molecular signatures in hematological malignancies. PloS one 9: e106788 doi: 10.1371/journal.pone.0106788 25207815 10.1371/journal.pone.0106788 PMC4160201

[47] Lupey-Green LN, Moquin SA, Martin KA, McDevitt SM, Hulse M, et al (2017) PARP1 restricts Epstein Barr Virus lytic reactivation by binding the BZLF1 promoter. Virology 507: 220–230. doi: 10.1016/j.virol.2017.04.006 28456021 10.1016/j.virol.2017.04.006 PMC5521201

[48] Hu X, Yang D, Zimmerman M, Liu F, Yang J, et al (2011) IRF8 regulates acid ceramidase expression to mediate apoptosis and suppresses myelogeneous leukemia. Cancer Res 71: 2882–2891. doi: 10.1158/0008-5472.CAN-10-2493 21487040 10.1158/0008-5472.CAN-10-2493 PMC3078194

[49] Yang D, Thangaraju M, Browning DD, Dong Z, Korchin B, et al (2007) IFN regulatory factor 8 mediates apoptosis in nonhemopoietic tumor cells via regulation of Fas expression. J Immunol 179: 4775–4782. 17878376 10.4049/jimmunol.179.7.4775

[50] Yang D, Wang S, Brooks C, Dong Z, Schoenlein PV, et al (2009) IFN regulatory factor 8 sensitizes soft tissue sarcoma cells to death receptor-initiated apoptosis via repression of FLICE-like protein expression. Cancer Res 69: 1080–1088. doi: 10.1158/0008-5472.CAN-08-2520 19155307 10.1158/0008-5472.CAN-08-2520 PMC2633427

[51] Prasad A, Remick J, Zeichner SL (2013) Activation of human herpesvirus replication by apoptosis. J Virol 87: 10641–10650. doi: 10.1128/JVI.01178-13 23885073 10.1128/JVI.01178-13 PMC3807386

[52] Kim H, Choi H, Lee SK (2015) Epstein-Barr Virus MicroRNA miR-BART20-5p Suppresses Lytic Induction by Inhibiting BAD-Mediated caspase-3-Dependent Apoptosis. J Virol 90: 1359–1368. doi: 10.1128/JVI.02794-15 26581978 10.1128/JVI.02794-15 PMC4719597

[53] Kenney SC, Mertz JE (2014) Regulation of the latent-lytic switch in Epstein-Barr virus. Semin Cancer Biol 26: 60–68. doi: 10.1016/j.semcancer.2014.01.002 24457012 10.1016/j.semcancer.2014.01.002 PMC4048781

[54] Shimbo K, Hsu GW, Nguyen H, Mahrus S, Trinidad JC, et al (2012) Quantitative profiling of caspase-cleaved substrates reveals different drug-induced and cell-type patterns in apoptosis. Proc Natl Acad Sci U S A 109: 12432–12437. doi: 10.1073/pnas.1208616109 22802652 10.1073/pnas.1208616109 PMC3412033

[55] Julien O, Zhuang M, Wiita AP, O’Donoghue AJ, Knudsen GM, et al (2016) Quantitative MS-based enzymology of caspases reveals distinct protein substrate specificities, hierarchies, and cellular roles. Proc Natl Acad Sci U S A 113: E2001–2010. doi: 10.1073/pnas.1524900113 27006500 10.1073/pnas.1524900113 PMC4833263

[56] Julien O, Wells JA (2017) Caspases and their substrates. Cell Death Differ 24: 1380–1389. doi: 10.1038/cdd.2017.44 28498362 10.1038/cdd.2017.44 PMC5520456

[57] Gjyshi O, Roy A, Dutta S, Veettil MV, Dutta D, et al (2015) Activated Nrf2 Interacts with Kaposi’s Sarcoma-Associated Herpesvirus Latency Protein LANA-1 and Host Protein KAP1 To Mediate Global Lytic Gene Repression. J Virol 89: 7874–7892. doi: 10.1128/JVI.00895-15 25995248 10.1128/JVI.00895-15 PMC4505678

[58] Rauwel B, Jang SM, Cassano M, Kapopoulou A, Barde I, et al (2015) Release of human cytomegalovirus from latency by a KAP1/TRIM28 phosphorylation switch. Elife 4. 10.7554/eLife.06068 PMC4384640 25846574

[59] Sun R, Liang D, Gao Y, Lan K (2014) Kaposi’s sarcoma-associated herpesvirus-encoded LANA interacts with host KAP1 to facilitate establishment of viral latency. J Virol 88: 7331–7344. doi: 10.1128/JVI.00596-14 24741090 10.1128/JVI.00596-14 PMC4054432

[60] Lee N, Yario TA, Gao JS, Steitz JA (2016) EBV noncoding RNA EBER2 interacts with host RNA-binding proteins to regulate viral gene expression. Proc Natl Acad Sci U S A 113: 3221–3226. doi: 10.1073/pnas.1601773113 26951683 10.1073/pnas.1601773113 PMC4812724

[61] Lee N, Moss WN, Yario TA, Steitz JA (2015) EBV noncoding RNA binds nascent RNA to drive host PAX5 to viral DNA. Cell 160: 607–618. doi: 10.1016/j.cell.2015.01.015 25662012 10.1016/j.cell.2015.01.015 PMC4329084

[62] Arvey A, Tempera I, Tsai K, Chen HS, Tikhmyanova N, et al (2012) An atlas of the Epstein-Barr virus transcriptome and epigenome reveals host-virus regulatory interactions. Cell Host Microbe 12: 233–245. doi: 10.1016/j.chom.2012.06.008 22901543 10.1016/j.chom.2012.06.008 PMC3424516

[63] Gray KS, Forrest JC, Speck SH (2010) The de novo methyltransferases DNMT3a and DNMT3b target the murine gammaherpesvirus immediate-early gene 50 promoter during establishment of latency. J Virol 84: 4946–4959. doi: 10.1128/JVI.00060-10 20200245 10.1128/JVI.00060-10 PMC2863815

[64] Li X, Bhaduri-McIntosh S (2016) A Central Role for STAT3 in Gammaherpesvirus-Life Cycle and -Diseases. Front Microbiol 7: 1052 doi: 10.3389/fmicb.2016.01052 27458446 10.3389/fmicb.2016.01052 PMC4937026

[65] King CA, Li X, Barbachano-Guerrero A, Bhaduri-McIntosh S (2015) STAT3 Regulates Lytic Activation of Kaposi’s Sarcoma-Associated Herpesvirus. J Virol 89: 11347–11355. doi: 10.1128/JVI.02008-15 26339061 10.1128/JVI.02008-15 PMC4645641

[66] Koganti S, Clark C, Zhi J, Li X, Chen EI, et al (2015) Cellular STAT3 functions via PCBP2 to restrain Epstein-Barr Virus lytic activation in B lymphocytes. J Virol 89: 5002–5011. doi: 10.1128/JVI.00121-15 25717101 10.1128/JVI.00121-15 PMC4403452

[67] Hill ER, Koganti S, Zhi J, Megyola C, Freeman AF, et al (2013) Signal transducer and activator of transcription 3 limits Epstein-Barr virus lytic activation in B lymphocytes. J Virol 87: 11438–11446. doi: 10.1128/JVI.01762-13 23966384 10.1128/JVI.01762-13 PMC3807321

[68] Daigle D, Megyola C, El-Guindy A, Gradoville L, Tuck D, et al (2010) Upregulation of STAT3 marks Burkitt lymphoma cells refractory to Epstein-Barr virus lytic cycle induction by HDAC inhibitors. J Virol 84: 993–1004. doi: 10.1128/JVI.01745-09 19889776 10.1128/JVI.01745-09 PMC2798381

[69] Shin DM, Lee CH, Morse HC 3rd (2011) IRF8 governs expression of genes involved in innate and adaptive immunity in human and mouse germinal center B cells. PLoS One 6: e27384 doi: 10.1371/journal.pone.0027384 22096565 10.1371/journal.pone.0027384 PMC3214047

[70] Sciammas R, Shaffer AL, Schatz JH, Zhao H, Staudt LM, et al (2006) Graded expression of interferon regulatory factor-4 coordinates isotype switching with plasma cell differentiation. Immunity 25: 225–236. doi: 10.1016/j.immuni.2006.07.009 16919487 10.1016/j.immuni.2006.07.009

[71] Salem S, Langlais D, Lefebvre F, Bourque G, Bigley V, et al (2014) Functional characterization of the human dendritic cell immunodeficiency associated with the IRF8(K108E) mutation. Blood 124: 1894–1904. doi: 10.1182/blood-2014-04-570879 25122610 10.1182/blood-2014-04-570879 PMC4168344

[72] Calattini S, Sereti I, Scheinberg P, Kimura H, Childs RW, et al (2010) Detection of EBV genomes in plasmablasts/plasma cells and non-B cells in the blood of most patients with EBV lymphoproliferative disorders by using Immuno-FISH. Blood 116: 4546–4559. doi: 10.1182/blood-2010-05-285452 20699441 10.1182/blood-2010-05-285452 PMC2996115

[73] Mboko WP, Rekow MM, Ledwith MP, Lange PT, Schmitz KE, et al (2017) Interferon Regulatory Factor 1 and Type I Interferon Cooperate To Control Acute Gammaherpesvirus Infection. J Virol 91. 10.1128/JVI.01444-16 PMC5165188 27795415

[74] Mboko WP, Olteanu H, Ray A, Xin G, Darrah EJ, et al (2015) Tumor Suppressor Interferon-Regulatory Factor 1 Counteracts the Germinal Center Reaction Driven by a Cancer-Associated Gammaherpesvirus. J Virol 90: 2818–2829. doi: 10.1128/JVI.02774-15 26719266 10.1128/JVI.02774-15 PMC4810652

[75] Mboko WP, Mounce BC, Emmer J, Darrah E, Patel SB, et al (2014) Interferon regulatory factor 1 restricts gammaherpesvirus replication in primary immune cells. J Virol 88: 6993–7004. doi: 10.1128/JVI.00638-14 24719409 10.1128/JVI.00638-14 PMC4054362

[76] Dutia BM, Allen DJ, Dyson H, Nash AA (1999) Type I interferons and IRF-1 play a critical role in the control of a gammaherpesvirus infection. Virology 261: 173–179. doi: 10.1006/viro.1999.9834 10497103 10.1006/viro.1999.9834

[77] Mandal P, Krueger BE, Oldenburg D, Andry KA, Beard RS, et al (2011) A gammaherpesvirus cooperates with interferon-alpha/beta-induced IRF2 to halt viral replication, control reactivation, and minimize host lethality. PLoS Pathog 7: e1002371 doi: 10.1371/journal.ppat.1002371 22114555 10.1371/journal.ppat.1002371 PMC3219715

[78] Hwang S, Kim KS, Flano E, Wu TT, Tong LM, et al (2009) Conserved herpesviral kinase promotes viral persistence by inhibiting the IRF-3-mediated type I interferon response. Cell Host Microbe 5: 166–178. doi: 10.1016/j.chom.2008.12.013 19218087 10.1016/j.chom.2008.12.013 PMC2749518

[79] Bi X, Yang L, Mancl ME, Barnes BJ (2011) Modulation of interferon regulatory factor 5 activities by the Kaposi sarcoma-associated herpesvirus-encoded viral interferon regulatory factor 3 contributes to immune evasion and lytic induction. J Interferon Cytokine Res 31: 373–382. doi: 10.1089/jir.2010.0084 21133648 10.1089/jir.2010.0084

[80] Liang Q, Fu B, Wu F, Li X, Yuan Y, et al (2012) ORF45 of Kaposi’s sarcoma-associated herpesvirus inhibits phosphorylation of interferon regulatory factor 7 by IKKepsilon and TBK1 as an alternative substrate. J Virol 86: 10162–10172. doi: 10.1128/JVI.05224-11 22787218 10.1128/JVI.05224-11 PMC3446610

[81] Hwang SW, Kim D, Jung JU, Lee HR (2017) KSHV-encoded viral interferon regulatory factor 4 (vIRF4) interacts with IRF7 and inhibits interferon alpha production. Biochem Biophys Res Commun 486: 700–705. doi: 10.1016/j.bbrc.2017.03.101 28342865 10.1016/j.bbrc.2017.03.101 PMC5490377

[82] Forero A, McCormick KD, Jenkins FJ, Sarkar SN (2014) Downregulation of IRF4 induces lytic reactivation of KSHV in primary effusion lymphoma cells. Virology 458–459: 4–10. doi: 10.1016/j.virol.2014.04.020 24928034 10.1016/j.virol.2014.04.020 PMC4058074

[83] Lee HR, Doganay S, Chung B, Toth Z, Brulois K, et al (2014) Kaposi’s sarcoma-associated herpesvirus viral interferon regulatory factor 4 (vIRF4) targets expression of cellular IRF4 and the Myc gene to facilitate lytic replication. J Virol 88: 2183–2194. doi: 10.1128/JVI.02106-13 24335298 10.1128/JVI.02106-13 PMC3911525

[84] Forero A, Moore PS, Sarkar SN (2013) Role of IRF4 in IFN-stimulated gene induction and maintenance of Kaposi sarcoma-associated herpesvirus latency in primary effusion lymphoma cells. J Immunol 191: 1476–1485. doi: 10.4049/jimmunol.1202514 23804715 10.4049/jimmunol.1202514 PMC3740746

[85] Matar CG, Rangaswamy US, Wakeman BS, Iwakoshi N, Speck SH (2014) Murine gammaherpesvirus 68 reactivation from B cells requires IRF4 but not XBP-1. J Virol 88: 11600–11610. doi: 10.1128/JVI.01876-14 25078688 10.1128/JVI.01876-14 PMC4178818

[86] Rangaswamy US, Speck SH (2014) Murine gammaherpesvirus M2 protein induction of IRF4 via the NFAT pathway leads to IL-10 expression in B cells. PLoS Pathog 10: e1003858 doi: 10.1371/journal.ppat.1003858 24391506 10.1371/journal.ppat.1003858 PMC3879372

[87] Ersing I, Nobre L, Wang LW, Soday L, Ma Y, et al (2017) A Temporal Proteomic Map of Epstein-Barr Virus Lytic Replication in B Cells. Cell Rep 19: 1479–1493. doi: 10.1016/j.celrep.2017.04.062 28514666 10.1016/j.celrep.2017.04.062 PMC5446956

[88] Xu H, Chaudhri VK, Wu Z, Biliouris K, Dienger-Stambaugh K, et al (2015) Regulation of bifurcating B cell trajectories by mutual antagonism between transcription factors IRF4 and IRF8. Nat Immunol 16: 1274–1281. doi: 10.1038/ni.3287 26437243 10.1038/ni.3287

[89] Pathak S, Ma S, Shukla V, Lu R (2013) A role for IRF8 in B cell anergy. J Immunol 191: 6222–6230. doi: 10.4049/jimmunol.1301169 24218455 10.4049/jimmunol.1301169 PMC3864091

[90] Gastaldello S, Chen X, Callegari S, Masucci MG (2013) Caspase-1 promotes Epstein-Barr virus replication by targeting the large tegument protein deneddylase to the nucleus of productively infected cells. PLoS Pathog 9: e1003664 doi: 10.1371/journal.ppat.1003664 24130483 10.1371/journal.ppat.1003664 PMC3795028

[91] Agard NJ, Maltby D, Wells JA (2010) Inflammatory stimuli regulate caspase substrate profiles. Mol Cell Proteomics 9: 880–893. doi: 10.1074/mcp.M900528-MCP200 20173201 10.1074/mcp.M900528-MCP200 PMC2871421

[92] De Leo A, Chen HS, Hu CA, Lieberman PM (2017) Deregulation of KSHV latency conformation by ER-stress and caspase-dependent RAD21-cleavage. PLoS Pathog 13: e1006596 doi: 10.1371/journal.ppat.1006596 28854249 10.1371/journal.ppat.1006596 PMC5595345

[93] Denes A, Lopez-Castejon G, Brough D (2012) Caspase-1: is IL-1 just the tip of the ICEberg? Cell Death Dis 3: e338 doi: 10.1038/cddis.2012.86 22764097 10.1038/cddis.2012.86 PMC3406585

[94] Zhang K, Lv D-W, Li R (2017) B Cell Receptor Activation and Chemical Induction Trigger Caspase-Mediated Cleavage of PIAS1 to Facilitate Epstein-Barr Virus Reactivation. Cell Rep 21: 3445–3457. doi: 10.1016/j.celrep.2017.11.071 29262325 10.1016/j.celrep.2017.11.071 PMC5741098

[95] Togi S, Hatano Y, Muromoto R, Kawanishi E, Ikeda O, et al (2016) Caspase-dependent cleavage regulates protein levels of Epstein-Barr virus-derived latent membrane protein 1. FEBS Lett 590: 808–818. doi: 10.1002/1873-3468.12119 26921582 10.1002/1873-3468.12119

[96] Backes C, Kuentzer J, Lenhof HP, Comtesse N, Meese E (2005) GraBCas: a bioinformatics tool for score-based prediction of Caspase- and Granzyme B-cleavage sites in protein sequences. Nucleic Acids Res 33: W208–213. doi: 10.1093/nar/gki433 15980455 10.1093/nar/gki433 PMC1160194

[97] Wilkins MR, Gasteiger E, Bairoch A, Sanchez JC, Williams KL, et al (1999) Protein identification and analysis tools in the ExPASy server. Methods Mol Biol 112: 531–552. 10027275 10.1385/1-59259-584-7:531

[98] Zhang L, Zhu C, Guo Y, Wei F, Lu J, et al (2014) Inhibition of KAP1 enhances hypoxia-induced Kaposi’s sarcoma-associated herpesvirus reactivation through RBP-Jkappa. J Virol 88: 6873–6884. doi: 10.1128/JVI.00283-14 24696491 10.1128/JVI.00283-14 PMC4054365

[99] Liao G, Huang J, Fixman ED, Hayward SD (2005) The Epstein-Barr virus replication protein BBLF2/3 provides an origin-tethering function through interaction with the zinc finger DNA binding protein ZBRK1 and the KAP-1 corepressor. J Virol 79: 245–256. doi: 10.1128/JVI.79.1.245-256.2005 15596820 10.1128/JVI.79.1.245-256.2005 PMC538732

[100] Hauser J, Verma-Gaur J, Wallenius A, Grundstrom T (2009) Initiation of antigen receptor-dependent differentiation into plasma cells by calmodulin inhibition of E2A. J Immunol 183: 1179–1187. doi: 10.4049/jimmunol.0900455 19553523 10.4049/jimmunol.0900455

[101] Carotta S, Willis SN, Hasbold J, Inouye M, Pang SH, et al (2014) The transcription factors IRF8 and PU.1 negatively regulate plasma cell differentiation. J Exp Med 211: 2169–2181. doi: 10.1084/jem.20140425 25288399 10.1084/jem.20140425 PMC4203955

[102] Sun L, St Leger AJ, Yu CR, He C, Mahdi RM, et al (2016) Interferon Regulator Factor 8 (IRF8) Limits Ocular Pathology during HSV-1 Infection by Restraining the Activation and Expansion of CD8+ T Cells. PLoS One 11: e0155420 doi: 10.1371/journal.pone.0155420 27171004 10.1371/journal.pone.0155420 PMC4865128

[103] Ayithan N, Bradfute SB, Anthony SM, Stuthman KS, Bavari S, et al (2015) Virus-like particles activate type I interferon pathways to facilitate post-exposure protection against Ebola virus infection. PLoS One 10: e0118345 doi: 10.1371/journal.pone.0118345 25719445 10.1371/journal.pone.0118345 PMC4342244

[104] Tussiwand R, Everts B, Grajales-Reyes GE, Kretzer NM, Iwata A, et al (2015) Klf4 expression in conventional dendritic cells is required for T helper 2 cell responses. Immunity 42: 916–928. doi: 10.1016/j.immuni.2015.04.017 25992862 10.1016/j.immuni.2015.04.017 PMC4447135

[105] Li R, Liao G, Nirujogi RS, Pinto SM, Shaw PG, et al (2015) Phosphoproteomic profiling reveals Epstein-Barr virus protein kinase integration of DNA damage response and mitotic signaling. PLoS Pathog 11: e1005346 doi: 10.1371/journal.ppat.1005346 26714015 10.1371/journal.ppat.1005346 PMC4699913

[106] Lv D-W, Zhong J, Zhang K, Pandey A, Li R (2017) Understanding Epstein-Barr Virus Life Cycle with Proteomics: A Temporal Analysis of Ubiquitination During Virus Reactivation. OMICS: A Journal of Integrative Biology 21: 27–37. doi: 10.1089/omi.2016.0158 28271981 10.1089/omi.2016.0158 PMC5240003

[107] Sarisky RT, Gao Z, Lieberman PM, Fixman ED, Hayward GS, et al (1996) A replication function associated with the activation domain of the Epstein-Barr virus Zta transactivator. Journal of virology 70: 8340–8347. 8970953 10.1128/jvi.70.12.8340-8347.1996 PMC190921

[108] Sanjana NE, Shalem O, Zhang F (2014) Improved vectors and genome-wide libraries for CRISPR screening. Nature methods 11: 783–784. doi: 10.1038/nmeth.3047 25075903 10.1038/nmeth.3047 PMC4486245

[109] Carreira VS, Fan Y, Kurita H, Wang Q, Ko CI, et al (2015) Disruption of Ah Receptor Signaling during Mouse Development Leads to Abnormal Cardiac Structure and Function in the Adult. PLoS One 10: e0142440 doi: 10.1371/journal.pone.0142440 26555816 10.1371/journal.pone.0142440 PMC4640841

[110] Love MI, Huber W, Anders S (2014) Moderated estimation of fold change and dispersion for RNA-seq data with DESeq2. Genome Biol 15: 550 doi: 10.1186/s13059-014-0550-8 25516281 10.1186/s13059-014-0550-8 PMC4302049

[111] Wang J-T, Yang P-W, Lee C-P, Han C-H, Tsai C-H, et al (2005) Detection of Epstein–Barr virus BGLF4 protein kinase in virus replication compartments and virus particles. Journal of general virology 86: 3215–3225. doi: 10.1099/vir.0.81313-0 16298966 10.1099/vir.0.81313-0

