# Supplementary figures and images for: Interferon regulatory factor 8 regulates caspase-1 expression to facilitate Epstein-Barr virus reactivation in response to B cell receptor stimulation and chemical induction
Source: PLoS Pathog. 2018 Jan 22;14(1):e1006868. doi: 10.1371/journal.ppat.1006868 (PMC5794192; doi:10.1371/journal.ppat.1006868)

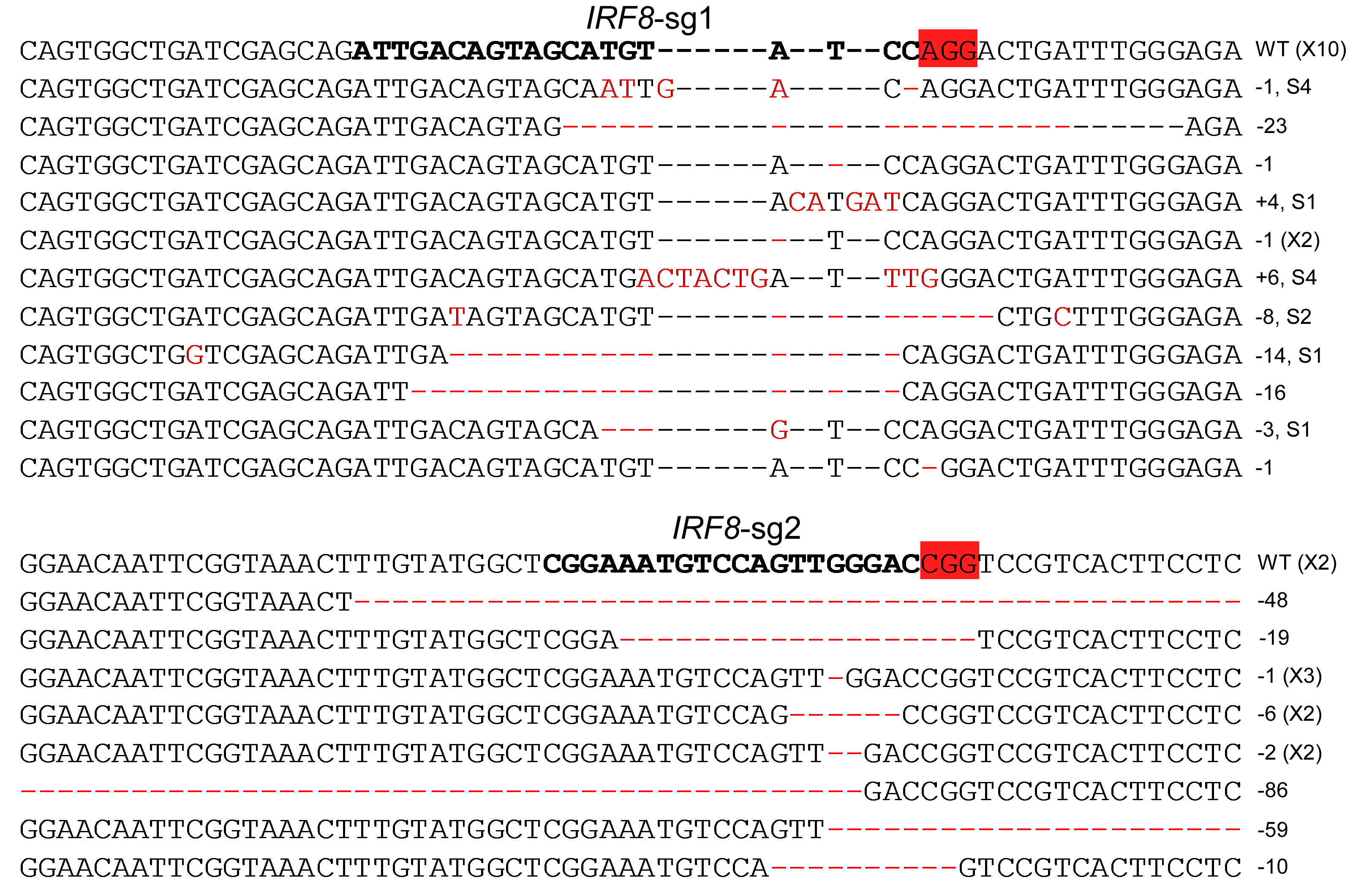

Supplement: S1 Fig — The sequencing of IRF8-depleted cell lines showing that 10 out of 22 clones for sg1 and 9 out of 14 clones for sg2 contain frame shifts. The PAM sequences were highlighted by red and the guide RNA sequences were shown in bold. WT: wild-type; “+” or “-” followed by numbers indicates the number of base pair inserted or deleted; “S” followed by numbers indicates the number of site mutations; “×” followed by numbers indicates the number of clones obtained in the sequencing. (TIF) [file ppat.1006868.s003.tif]

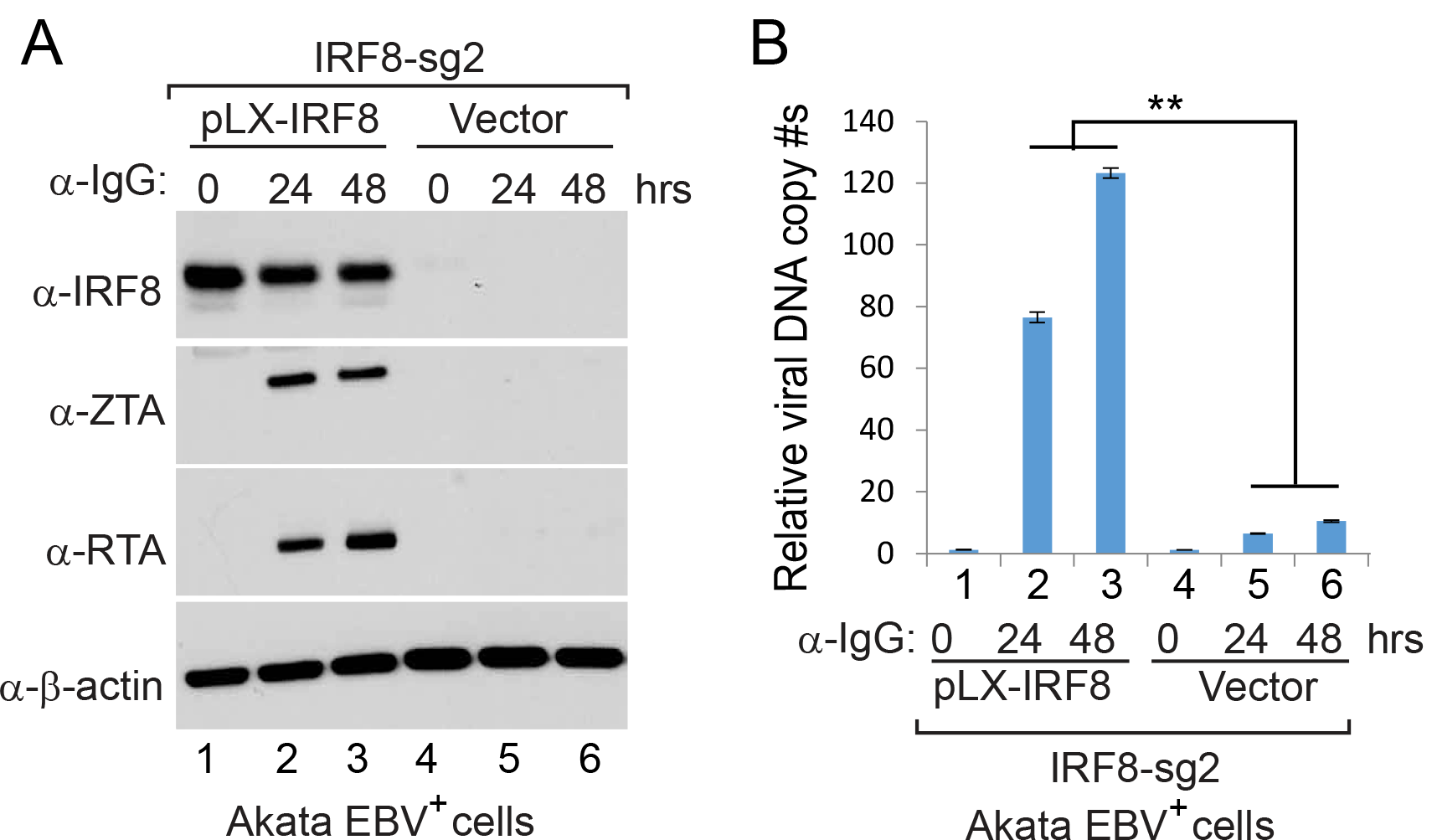

Supplement: S2 Fig — A. Akata (EBV+) IRF8-sg2 cells were used to establish IRF8-expressing stable cell lines using a pLX-IRF8 lentiviral construct. Western blot analyses showing IRF8, ZTA, RTA and BGLF4 expression level in different cell lines upon IgG cross-linking as indicated. B. Intracellular viral DNA from cells treated as in (A) was measured by qPCR using primers to EBV BALF5. The value of vector control at 0 hr (lane 4) was set as 1. Data are presented as means ± standard deviations of triplicate assays. ** p<0.01. (TIF) [file ppat.1006868.s004.tif]

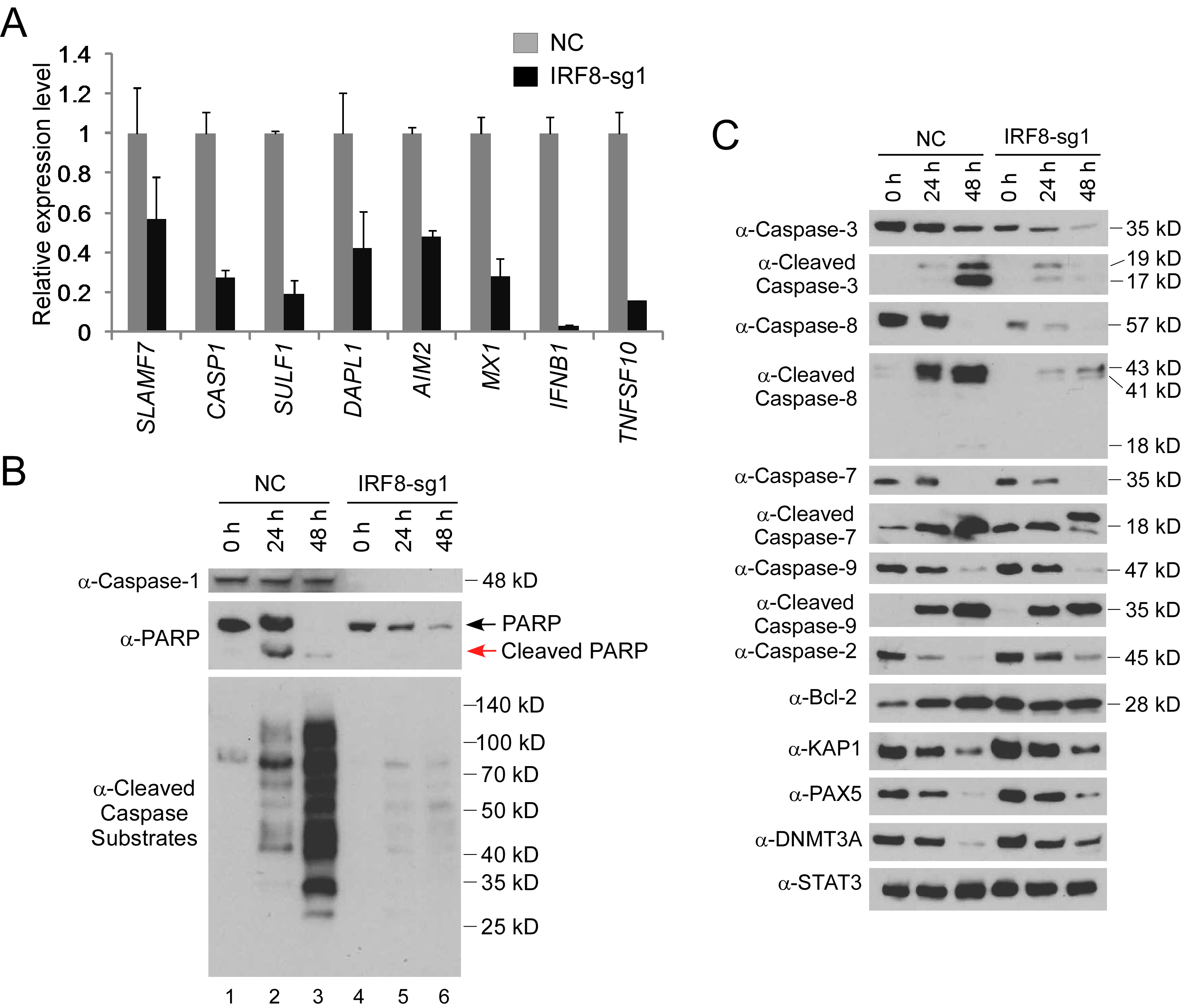

Supplement: S3 Fig — A. RT-qPCR validation of the 8 apoptosis-related genes in IRF8-sg1 cells. B and C. IRF8 depletion (sg1) suppresses caspase-1 expression and the generation of cleaved caspase substrates upon lytic induction by anti-IgG cross-linking. Western blot analysis of protein extracts from Fig 1C using antibodies against caspase-1, PARP, and cleaved caspase substrates as indicated in panel (B). Western blot analysis of protein extracts from Fig 1C using antibodies against caspase-3, cleaved caspase-3, caspase-8, cleaved caspase-8, caspase-7, cleaved caspase-7, caspase-9, cleaved caspass-9, caspase-2, Bcl2, KAP1, PAX5, DNMT3A and STAT3 as indicated in panel (C). (TIF) [file ppat.1006868.s005.tif]

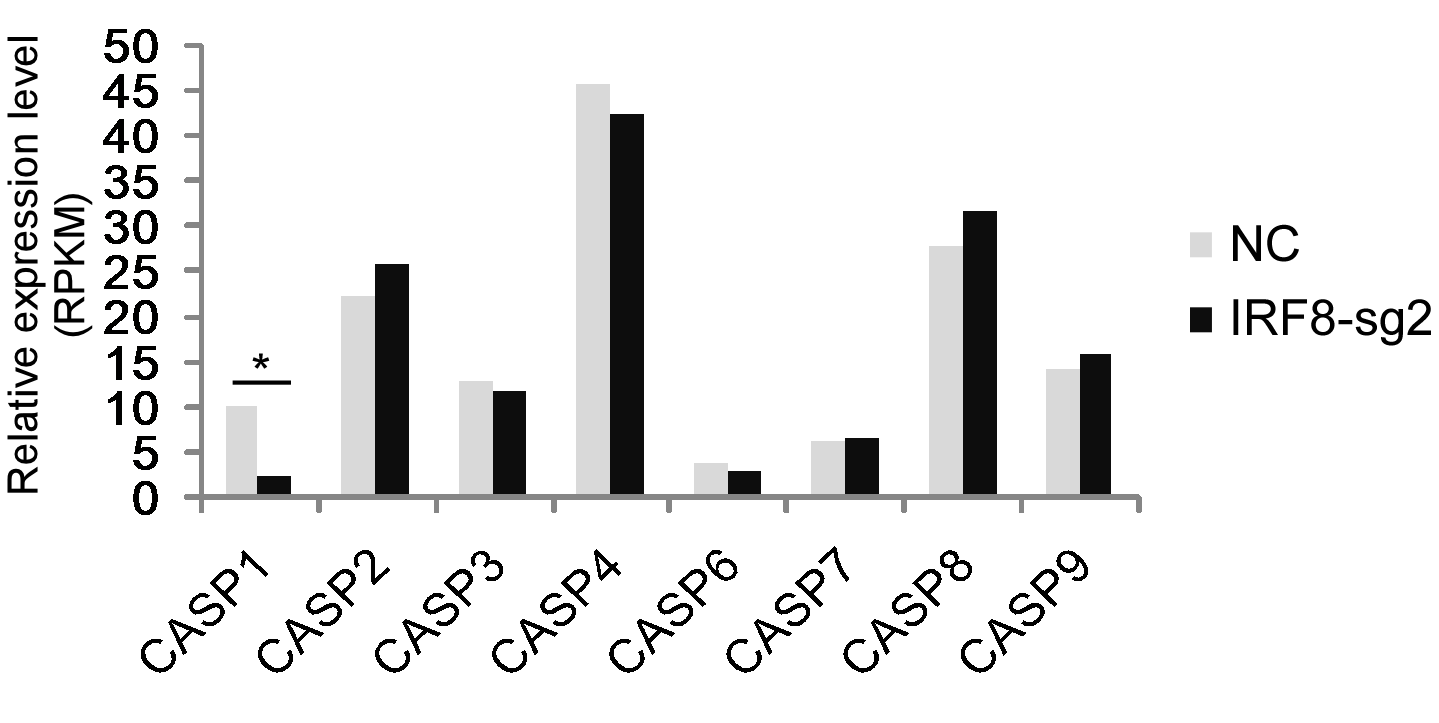

Supplement: S4 Fig — RPKM, Reads Per Kilobase of transcript per Million mapped reads. (TIF) [file ppat.1006868.s006.tif]

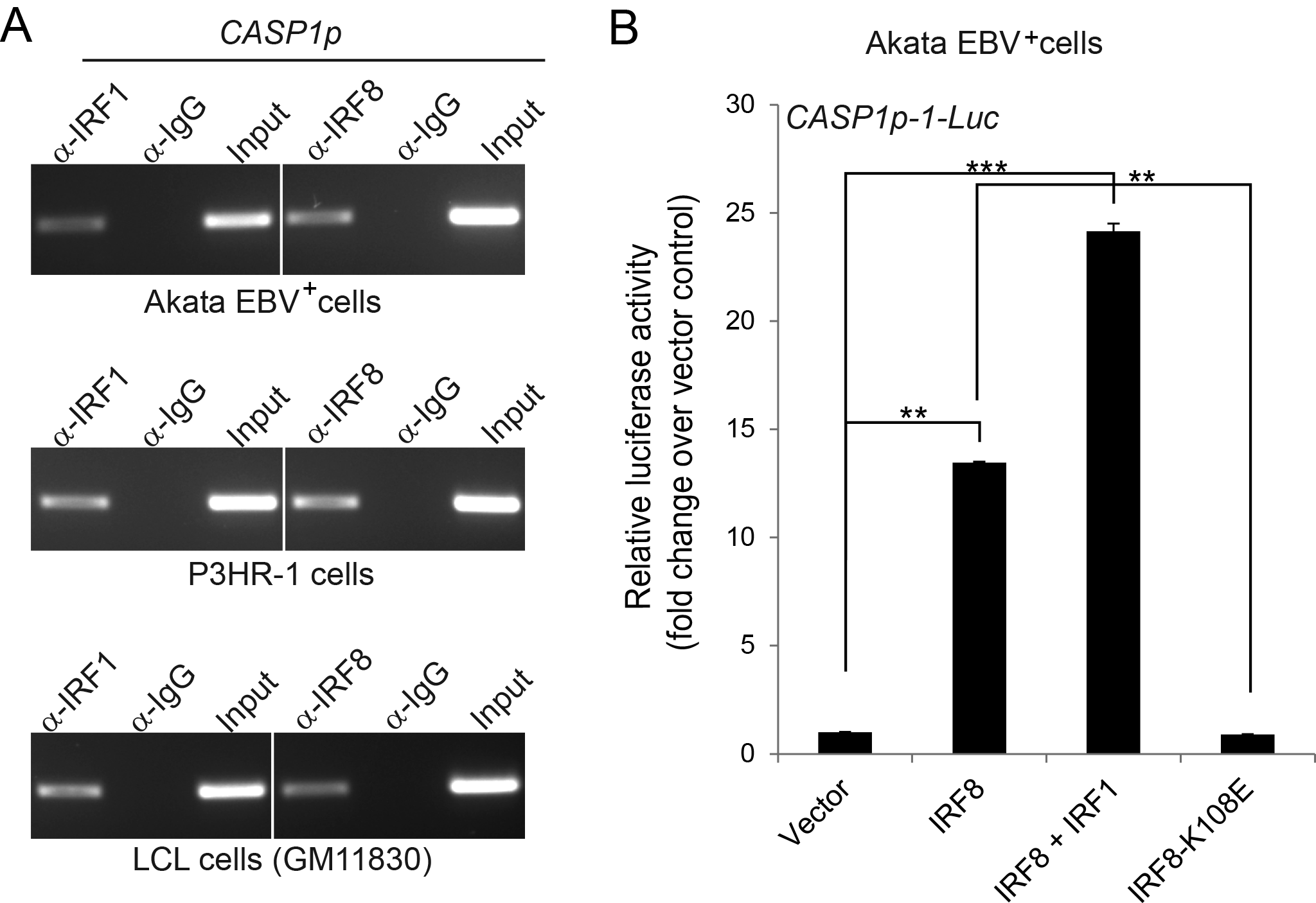

Supplement: S5 Fig — A. ChIP-PCR analysis using three EBV-positive cells [Akata (EBV+), P3HR-1 and LCLs] showing IRF8/IRF1 binding to CASP1 promoter. ChIP by a nonspecific IgG was include as negative controls. B. The pGL2-CASP1p-1-Luc constructs were co-transfected into Akata (EBV+) cells with either 10 ug of IRF8, IRF1 or IRF8-K108E expression vectors. Luciferase assays were performed 36 hrs post-transfection. The value of cells transfected with an empty vector was set as 1. The results were presented as mean ± standard deviation of triplicate assays. ** p<0.01, *** p<0.001. (TIF) [file ppat.1006868.s007.tif]

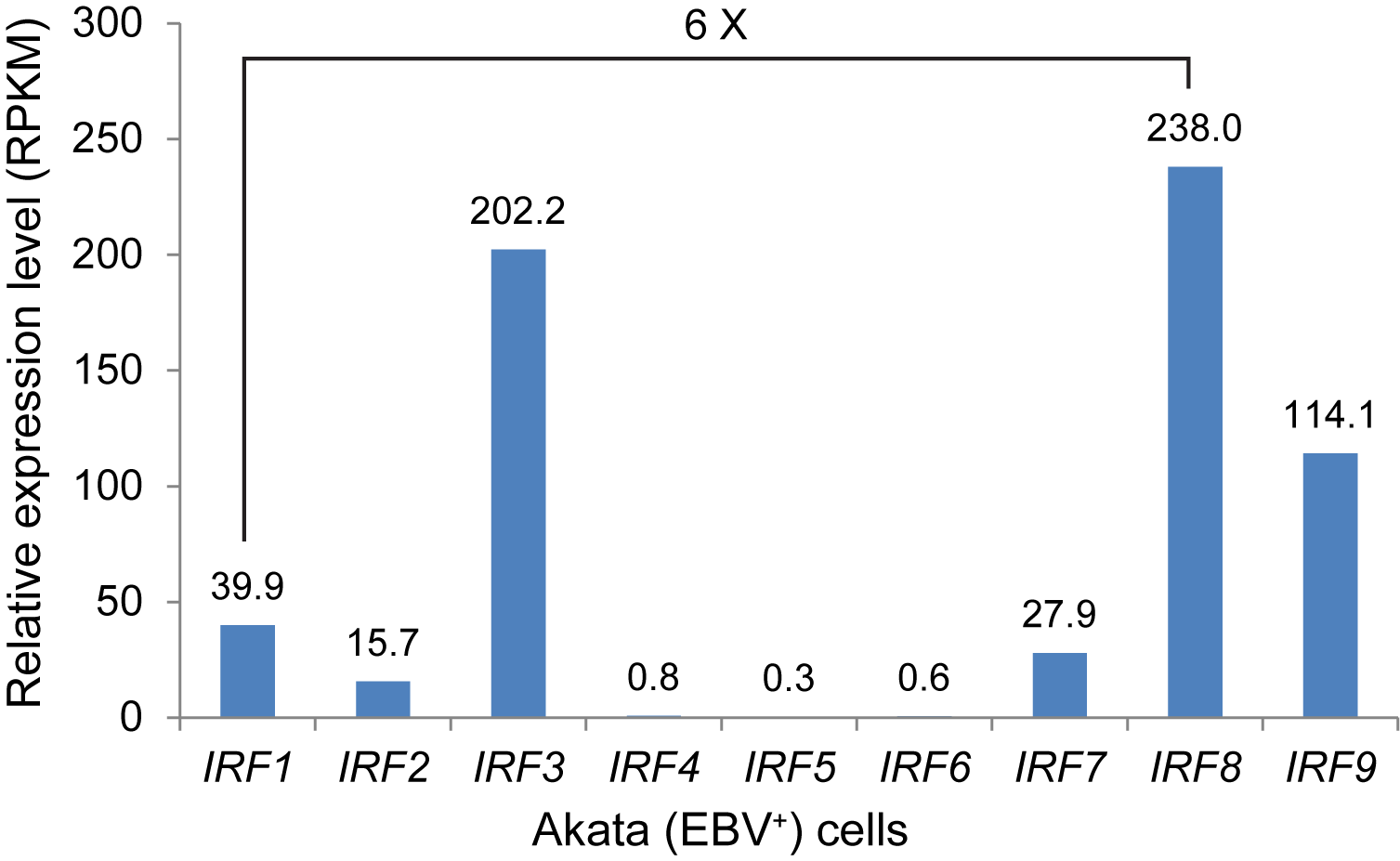

Supplement: S6 Fig — RPKM, Reads Per Kilobase of transcript per Million mapped reads. (TIF) [file ppat.1006868.s008.tif]

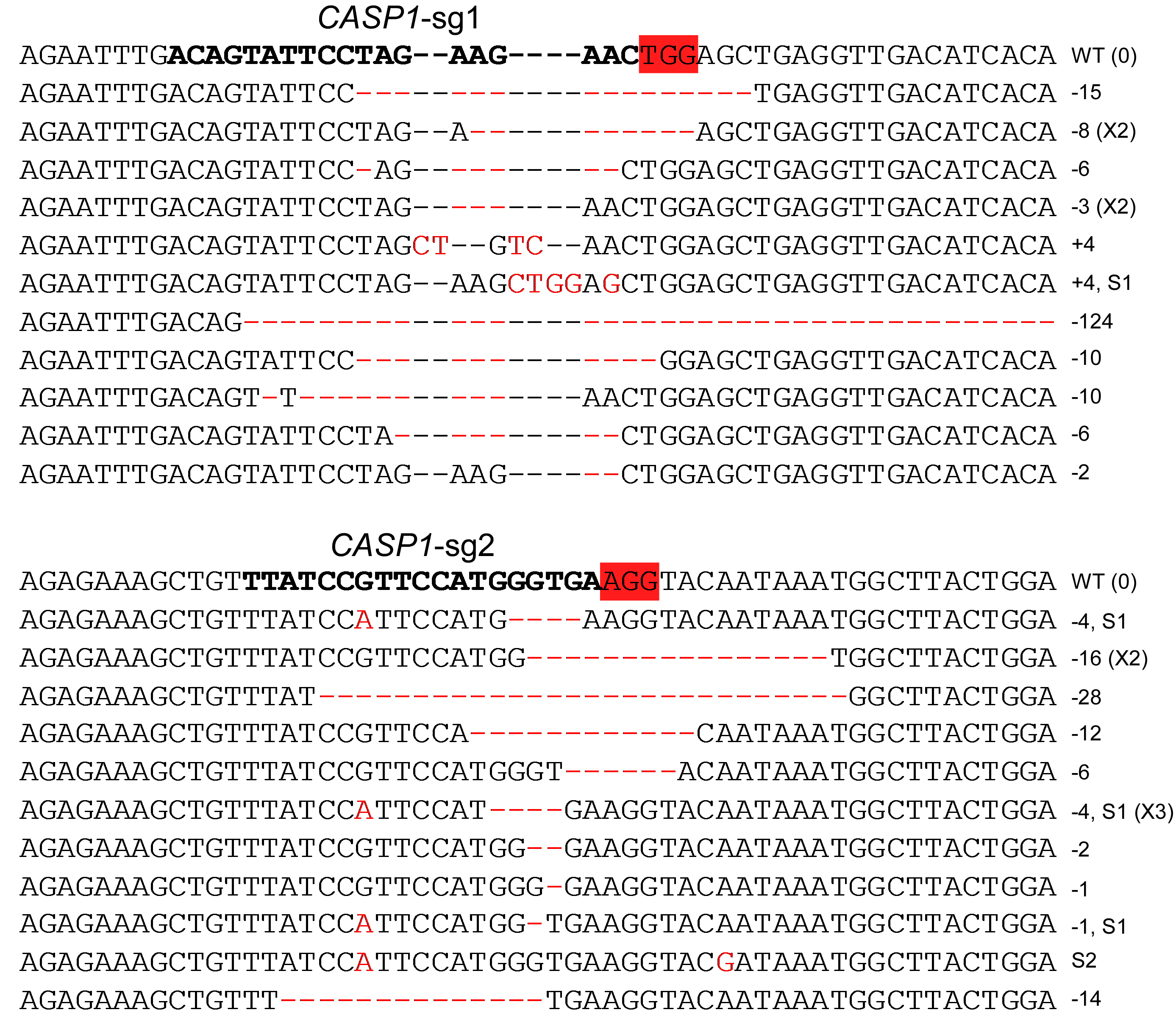

Supplement: S7 Fig — The sequencing of CASP1-depleted cell lines showing that 8 out of 13 clones for CASP1-sg1 and 12 out of 14 clones for CASP1-sg2 contain frame shifts. The PAM sequences were highlighted by red and the guide RNA sequences were shown in bold. WT: wild-type; “+” or “-” followed by numbers indicates the number of base pair inserted or deleted; “S” followed by numbers indicates the number of site mutations; “×” followed by numbers indicates the number of clones obtained in the sequencing. (TIF) [file ppat.1006868.s009.tif]

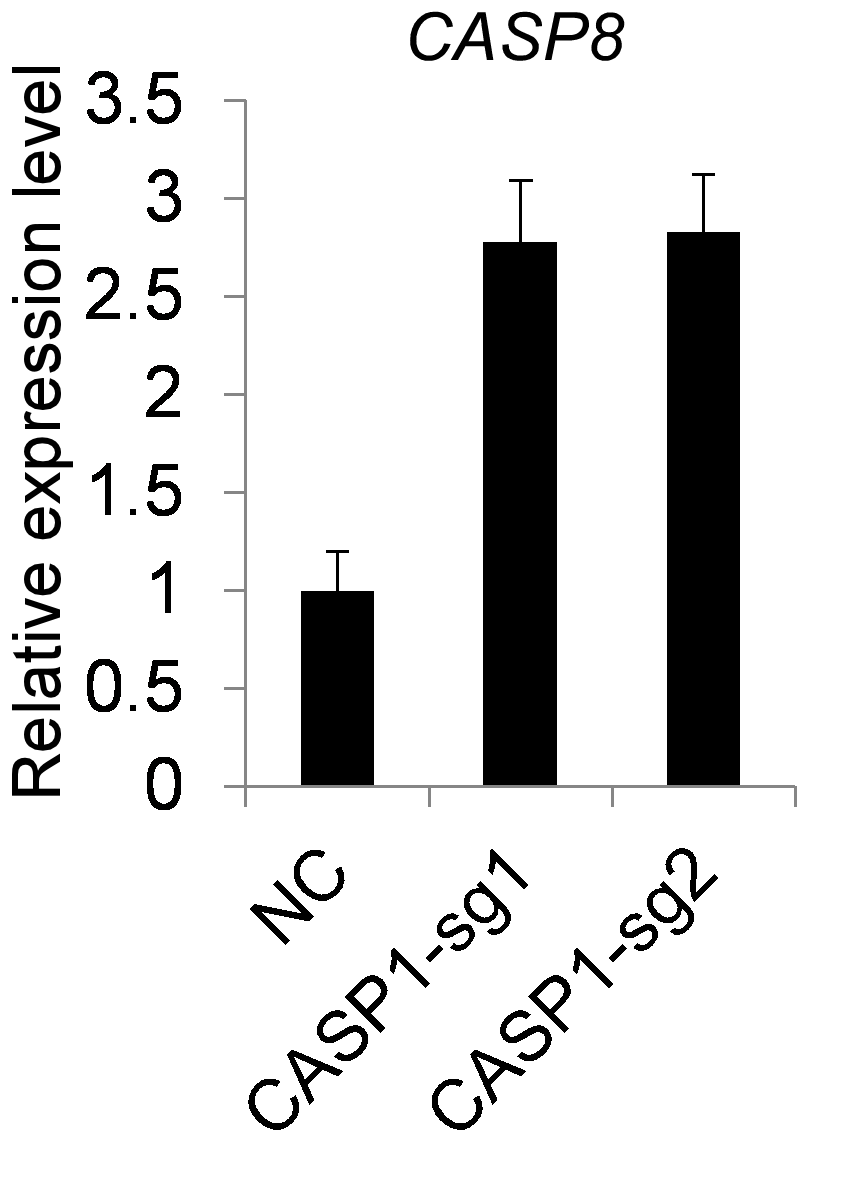

Supplement: S8 Fig — qPCR analysis showing that CASP8 mRNA level was slightly increased by CASP1 depletion. The value was normalized by qPCR using specific primers to β-actin. Data are presented as means ± standard deviations of triplicate assays. (TIF) [file ppat.1006868.s010.tif]
